# Boronate Esters Dynamic Networks for the Reduction of Mechanical Anisotropy in Vat 3D Printed Manufacts

**DOI:** 10.1021/acsapm.4c04101

**Published:** 2025-02-11

**Authors:** Alex Bonacini, Elena Saccani, Corrado Sciancalepore, Daniel Milanese, Gabriele Drago, Alessandro Pedrini, Roberta Pinalli, Renaud Nicolaÿ, Enrico Dalcanale

**Affiliations:** † Department of Chemistry, Life Sciences and Environmental Sustainability, University of Parma, Parco Area delle Scienze 17/A, 43124 Parma, Italy; ‡ Department of Systems and Industrial Technologies Engineering, University of Parma, Parco Area delle Scienze 181/A, 43124 Parma, Italy; § ELANTAS Europe S.r.l., Via San Martino 6, Alessandria 15028, Italy; ∥ Chimie Moléculaire, Macromoléculaire, Matériaux, ESPCI Paris, CNRS, Université PSL, 75005 Paris, France

**Keywords:** dynamic polymer networks, boronate esters, mechanical anisotropy, vat photopolymerization, 3D printing

## Abstract

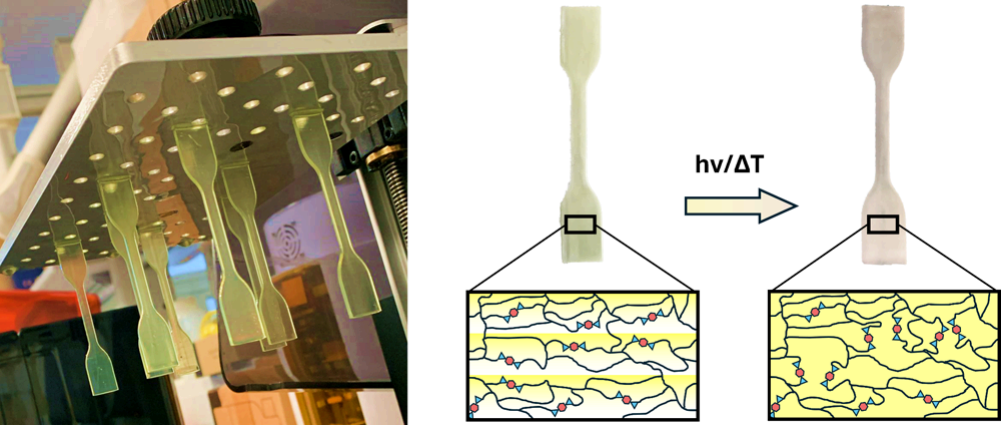

Vat photopolymerization (**VP**) is a prominent 3D printing technique known for its high resolution and precision. However, mechanical anisotropy can limit the performance of printed structures by making their mechanical properties dependent on the printing orientation and curing conditions. This study introduces a photocurable material for **VP** 3D printing, combining dynamic boronate ester-based cross-linking with nondynamic cross-links. The material is synthesized using photoinduced free radical polymerization of a (meth)acrylate-based formulation, incorporating a diboronate ester with two methacrylate functionalities (**DBEDMA**) and a commercial poly(propylene glycol) diacrylate (**PPGDA**). The resulting resins exhibit rapid curing kinetics, low shrinkage (5–8%), and tailored viscoelastic properties. Stress relaxation and creep recovery studies highlight the role of boronate ester metathesis in enabling network rearrangement and stress dissipation. The optimal formulation, **40D60P**, shows a significant reduction in mechanical anisotropy compared to an equivalent conventional resin containing only static cross-links (**40B60P**). Tensile tests confirm higher toughness and more consistent stress–strain behavior across printing orientations, attributed to partial topological rearrangement enabled by the dynamic cross-links. While improvements in isotropy are evident, a certain degree of mechanical anisotropy remains under specific conditions due to the presence of static cross-linking. Surface analysis via optical microscopy reveals smoother patterns in dynamic resin specimens, corroborating mechanical findings. This work demonstrates the potential of boronate ester-based dynamic chemistry to enhance the performance of **VP** 3D-printed materials, particularly in applications where reduced anisotropy and improved mechanical properties are critical.

## 1 Introduction

3D printing, also known as additive manufacturing (**AM**), provides efficient and cost-effective techniques for creating complex, customizable objects.^1^ Its versatility has broadened its applications across a wide range of fields, including aerospace,^2,3^ energy,^4^ construction,^5^ biomedicine,^6−11^ and many others.^12−18^ Among the various 3D printing methods, vat photopolymerization (**VP**) stands out for its ability to produce high-resolution structures using photopolymerizable resins. Key techniques, such as stereolithography (**SLA**), masked stereolithography (**MSLA**), and digital light processing (**DLP**), enable precise fabrication by selectively curing resin portions through localized exposure to UV–visible light.^19−21^

Despite these advantages, **VP** techniques have certain limitations, including relatively slow production times that increase with object size, the need for low-viscosity resins, and some degree of anisotropy in the mechanical properties of the final prints, influenced by factors like layer orientation, thickness, and irradiation time.^22−25^

Typical formulations for **VP** 3D printing consist of photopolymerizable oligomers and monomers combined with appropriate photoinitiators. Commonly employed polymerization mechanisms include free radical polymerization of (meth)acrylates,^26^ photoinduced radical thiol–ene click chemistry,^27^ and cationic ring-opening polymerization of epoxy groups.^28^

Recent advancements in **VP** 3D printable materials have focused on integrating specific functionalities into photopolymerizable formulations, resulting in materials with precise properties. One area of significant interest is the incorporation of dynamic covalent cross-links into **VP** 3D printable thermosets, enabling unique features such as postprinting reshaping, self-healing, adhesion, and reprocessing. Examples of dynamic covalent chemistries explored in **VP** 3D printing include β-hydroxy ester transesterification,^29,30^ disulfide exchange,^31,32^ and imine metathesis.^33^

A primary feature of materials that incorporate dynamic covalent cross-linking is their ability to relax applied stress by dissipating stored energy through topological reorganization. The relaxation rate is primarily influenced by the rate of exchange of dynamic bonds. In particular, materials containing boronate esters demonstrate the ability to rapidly relax stress at room temperature due to catalyst-free rapid metathesis reactions between dynamic covalent cross-links.^34−37^

The chemical compatibility of boronate esters with a wide range of functional groups and reactive species, such as (meth)acrylates, amines, imines, aldehydes, epoxides, thiols,^38,39^ and free radicals,^40,41^ is another highly attractive feature of these moieties, making them ideal candidates for **VP** 3D printing applications.

In 2021, Robinson et al. demonstrated the incorporation of dynamic boronate ester cross-links into **VP** 3D-printed materials using photoinduced radical thiol–ene addition. These materials exhibited covalently adaptable network properties, allowing for stress relaxation at room temperature, surface smoothing, and postprinting adhesion. Boronate ester metathesis was also used to functionalize the printed materials with dyes, enhancing the complexity of the final constructs. However, to prevent structural collapse at room temperature, a small percentage of nondynamic cross-links was required.^42^

In 2023, Sinawehl et al. developed boronate ester-based **VP** 3D-printed materials with potential applications in bone regeneration. By leveraging boronate ester chemistry, these materials could undergo controlled hydrolysis under both physiological and acidic conditions.^43^

**Figure 1:**
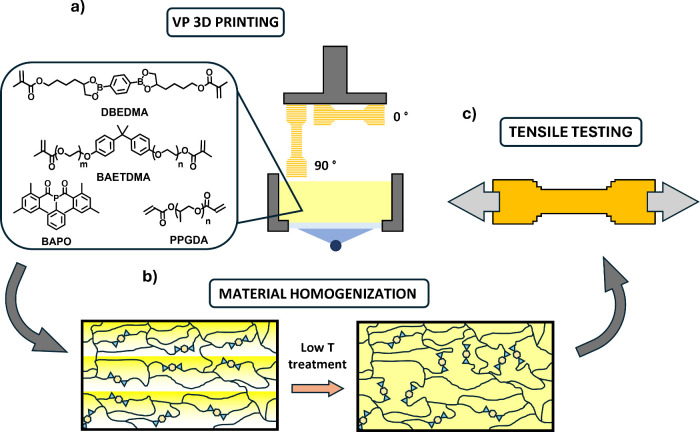
(a) 3D printing of photocurable resins in different orientations followed by (b) a low-temperature treatment to promote boronate ester metathesis reactions, (c) enhancing material mechanical isotropy evaluated through tensile tests.

In this work, we present a material obtained through photopolymerization that combines dynamic boronate ester-based cross-linking with nondynamic cross-linking. The material is synthesized via photoinduced free radical polymerization of a (meth)acrylate-based formulation and has demonstrated excellent suitability for **VP** 3D printing applications, exhibiting minimal shrinkage and rapid curing kinetics. Additionally, the material showed minimal creep at 65 °C, and 3D-printed specimens demonstrated reduced mechanical anisotropy compared to reference materials, when printed in different layer orientations. (Figure 1)

## 2 Experimental Section

### 2.1 Materials

The chemicals used for the synthesis were purchased from TCI, Sigma-Aldrich, Fisher, Alfa Aesar, and Acros Organics, and all reagents and solvents were used as received without further purification.

Poly(propylene glycol) diacrylate (**PPGDA**) with a reported average number of propylene glycol repeating units of 12 was purchased from TCI. Bisphenol A ethoxylate dimethacrylate (**BAETDMA**) was kindly provided by Elantas EUROPE S.r.l. (Collecchio, PR, Italy).

### 2.2 Synthesis of Diboronate Ester Dimethacrylate (DBEDMA)

4,5-Dihydroxypentyl methacrylate (compound **C** in Scheme S1), a key precursor for the synthesis of **DBEDMA**, was synthesized following the procedures outlined in the literature.^44^

**DBEDMA** was synthesized by reacting 1,4-phenylenediboronic acid (13.3 g, 80.0 mmol) with compound **C** (34.0 g, 168 mmol) in THF (173 mL) and water (0.54 mL). The mixture was stirred at room temperature for 10 min before adding anhydrous MgSO_4_ (57.8 g, 480 mmol). Stirring continued for 5 h at room temperature. The reaction mixture was then filtered, and the filtrate concentrated under reduced pressure, yielding **DBEDMA** as an orange oil (37.9 g, 76.1 mmol, 95% yield).

### 2.3 Preparation, Photocuring, 3D Printing and Postcuring of Photoresins

Photocurable formulations were prepared by mixing all the components in a light-protected container. All the mixtures were stirred for a minimum of 7 h while ensuring complete dissolution of the photo initiator.

Samples of photocured material used for Thermogravimetric Analysis (TGA), Differential Scanning Calorimetry (DSC), and Dynamic Mechanical Analysis (DMA) were prepared by irradiating a mold filled with the photocurable formulation for 5 min. The irradiation was carried out using a UV CHAMBER from AMS Technologies, equipped with a 365 nm monochromatic LED lamp that provided a power density of 55 mW/cm^2^.

3D printing was done using a Kentstrapper Aura stereolithography printer, which features a 4K UV LCD screen emitting monochromatic light at a wavelength of 405 nm. 3D printed tensile test samples were prepared in accordance with ISO 527-2, type 5A standards. All specimens were prepared with a printing speed of 15 s per layer, and with a layer thickness of 0.07 mm.

The postcuring of the 3D-printed specimens was performed using either a 365 nm monochromatic LED lamp from PHOTOELECTRONICS delivering a power density of 55 mW/cm^2^, or a Formlabs Form Cure, which has 13 LEDs with a power output of 39 W each and a wavelength of 405 nm.

### 2.4 Characterizations

NMR spectra, including ^1^H, COSY, ^13^C DEPT135, and HSQC, were recorded on a Bruker AVANCE 400 MHz or on a Jeol 600 MHz spectrometer using CDCl_3_ as the solvent.

Photo rheology experiments were performed using an Anton Paar MCR 102 rheometer. The device was equipped with an Omnicure Series 1500 lamp, which emits light in the 320–480 nm range. The power density at a wavelength of 365 nm was 5 mW/cm^2^. The experiments were conducted in air using an 8 mm disposable parallel plate geometry made of quartz glass, with a frequency of 1 Hz and a shear strain of 0.1%.

TGA were performed using a SETARAM THEMYS ONE instrument under a nitrogen atmosphere, with a heating rate of 10 °C/min.

DSC analyses were performed using a DSC250 instrument from the Discovery Series by TA Instruments, under air atmosphere, with a heating rate of 20 °C/min.

Images of the surface morphology of the 3D-printed specimens were captured using a Zeiss Axioskop optical microscope at 10× magnification.

DMA (1 Hz, 0.1% strain, heating rate of 3 °C/min), creep recovery and stress-relaxation analyses were conducted using a DMA Q800 instrument from TA Instruments.

Fourier transform infrared (FT-IR) spectroscopy analyses were performed using Bruker FTIR LUMOS.

The tensile tests were carried out using the TesT GmbH model 112 servomechanical machine in displacement control mode with a deformation rate of 1 mm/min.

## 3 Results and Discussion

The primary objective of this work is to develop a photocurable cross-linked material capable of rearranging its network topology at low temperatures by leveraging boronate ester metathesis chemistry. This strategy aims to mitigate the mechanical anisotropy commonly observed in 3D-printed structures. To effectively achieve this goal through moderate temperature post-treatments, it is essential to maintain a low glass transition temperature (*T*_g_). A low *T*_g_ ensures that the dynamic exchange of functionalities occurs without being restricted by the limited mobility of the polymer matrix.

### 3.1 Photoresins Formulation and Photocuring Study

**DBEDMA** was selected as the primary dynamic cross-linker and synthesized on a large scale (synthetic pathway detailed in Scheme S1, and NMR characterization provided in Figures S1–S5).

In addition to **DBEDMA**, a commercial poly(propylene glycol) diacrylate (**PPGDA**) was incorporated into the final photocurable formulations. **PPGDA** has an average molar mass of 851 g/mol, as determined by ^1^H NMR (refer to Figure S6, eqs S1 and S2). This long and flexible static cross-linker increases the overall flexibility of the material, allowing for network rearrangement while ensuring that the material retains its desired shape. The specific choice to combine a dimethacrylate-based cross-linker with a diacrylate-based cross-linker was driven by two main factors. First, a diacrylate-rich formulation was preferred to ensure the production of a low-Tg material, which is why **PPGDA** was selected. Second, the synthesis of a boronate ester diacrylate proved challenging, as one of the intermediates underwent polymerization during the purification process. Consequently, the corresponding dimethacrylate (**DBEDMA**) was chosen as an alternative. However, particular attention was given throughout the study to verifying the homogeneity of the obtained materials, which was primarily assessed using DMA.

A series of photocurable formulations with varying ratios of **DBEDMA** and **PPGDA** were tested. Each formulation contained the photoinitiator phenylbis(2,4,6-trimethylbenzoyl)phosphine oxide (**BAPO**) at a concentration of 1 mol % relative to the total moles of (meth)acrylate functions. This is equivalent to 2 mol % with respect to the total amount of cross-linker molecules. The molecular structures of the components are shown in Figure 1, and the molar ratios are specified in Table 1 (additional details in Table S1).

**Table 1:** Molar Ratios of the Components Used in the Photocurable Formulations; **DBEDMA** Is Indicated as **D**, **PPGDA** as **P**, and **BAETDMA** as **B**

formulation	DBEDMA	PPGDA	BAETDMA	BAPO (mol %)
**100P**	/	100 mol %	/	2
**10D90P**	10 mol %	90 mol %	/	2
**20D80P**	20 mol %	80 mol %	/	2
**30D70P**	30 mol %	70 mol %	/	2
**40D60P**	40 mol %	60 mol %	/	2
**60D40P**	60 mol %	40 mol %	/	2
80D20P	80 mol %	20 mol %	/	2
**100D**	100 mol %	/	/	2
**40B60P**	/	60 mol %	40 mol %	2

**Figure 2:**
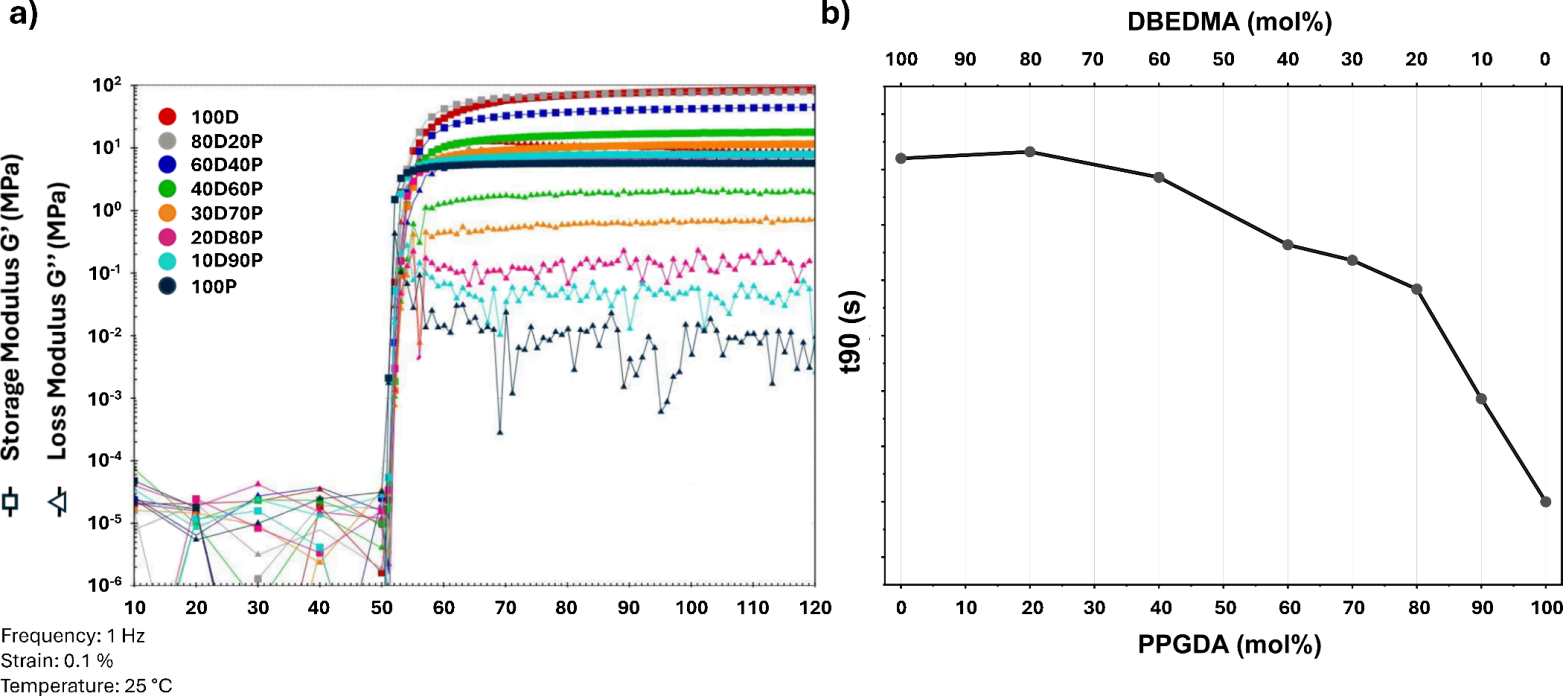
(a) *G*′ and *G*″ plotted as a function of time during the irradiation of the photoresins for 70 s, following a 50 s equilibration period in the dark, (b) corresponding t90 values, representing the time required to reach 90% of the final *G*′ value.

Preliminary studies of the viscoelastic response during photocuring were conducted using photorheology experiments at 25 °C. The results show a rapid increase in both storage modulus (*G*′) and loss modulus (*G*″) (Figure 2, left) immediately after the onset of irradiation at 50 s, with the moduli reaching a plateau within 40 s, depending on the specific formulation. Notably, a higher molar ratio of **DBEDMA** to **PPGDA** results in a slower photopolymerization rate, which reflects the lower reactivity of methacrylates compared to acrylates in radical polymerization. This slower reaction is clearly illustrated by the t90 values (Figure 2, right), representing the time required to reach 90% of the final *G*′ value, further reinforcing the observed trend.

Additionally, the final values of *G*′, *G*″, and complex viscosity |η*| (Figure S8) increase with the molar percentage of **DBEDMA** in the resin. This suggests that a higher **DBEDMA** content leads to a higher cross-linking density, due to the shorter molecular structure of **DBEDMA** compared to the longer **PPGDA**.

Finally, the postcuring shrinkage of the photoresins was measured by monitoring the thickness of the resin over time under a constant normal force applied by the rheometer.^45^ The results show minimal shrinkage, ranging from 5 to 8%, with no clear trend across the different formulations (Figure S9).

### 3.2 Photopolymerization and Viscoelastic Characterization of Materials

Flat samples were photocured into various shapes by irradiating resin-filled molds using a monochromatic 365 nm lamp for 5 min at a power density of 55 mW/cm^2^.

**Figure 3:**
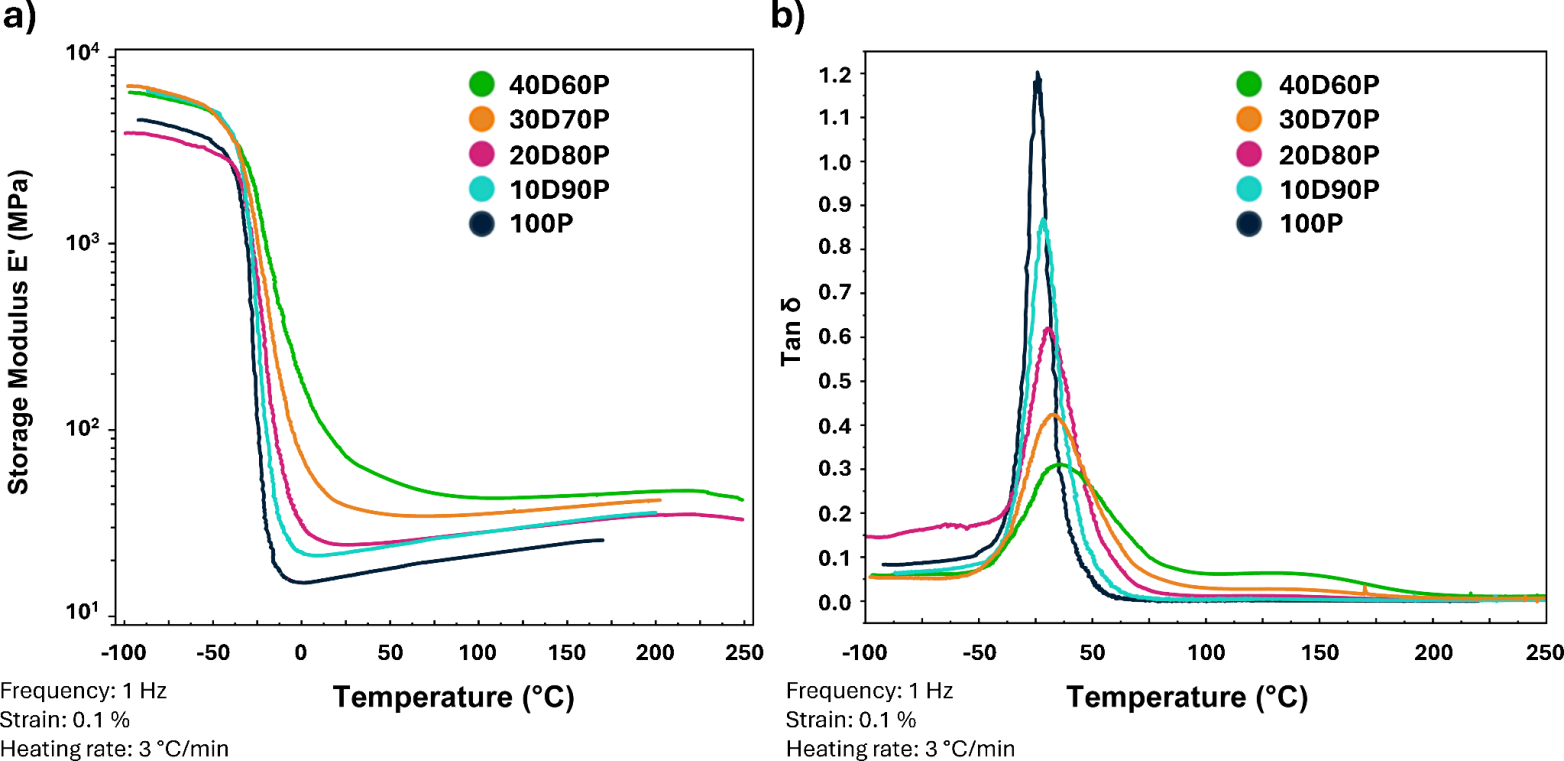
(a) Storage modulus *E*′ and (b) tan δ of photocured materials as a function of temperature.

The viscoelastic properties of the resulting materials, which remained thermally stable up to approximately 210 °C (Figure S10), were determined using DMA. DMA demonstrated that materials with **DBEDMA** content above 40 mol % exhibit heterogeneity, as evidenced by a broad tan δ peak (Figure S11). This prompted further investigations into formulations with a **DBEDMA** content cap at 40 mol %. This adjustment was made not only to improve material homogeneity but also to enable effective dilution of synthetic **DBEDMA** with commercially available molecules. The results (shown in Figure 3) indicate that all materials exhibit storage moduli (*E*′) in the range of 10^3^ MPa at low temperatures. Around −40 °C, the modulus decreases by 2 orders of magnitude, resulting in the formation of a rubbery plateau. The *E*′ values measured at 25 °C are consistent with the final *G*′ values obtained from photorheology, following the relationship *E*′ ≈ 3 *G*′. Moreover, the *E*′ values within the rubbery plateau exhibit the same trend observed in the photorheology experiments, increasing with the molar percentage of **DBEDMA**.

*T*_g_ were estimated using both DMA and DSC, with the values extrapolated from DMA based on tan δ peaks being higher than those obtained from the DSC curves (Figure S12). In both cases, the glass transition temperatures increased with the molar percentage of the more rigid **DBEDMA** relative to the more flexible **PPGDA**. However, all absolute *T*_g_ values were, as anticipated, lower than room temperature.

**Figure 4:**
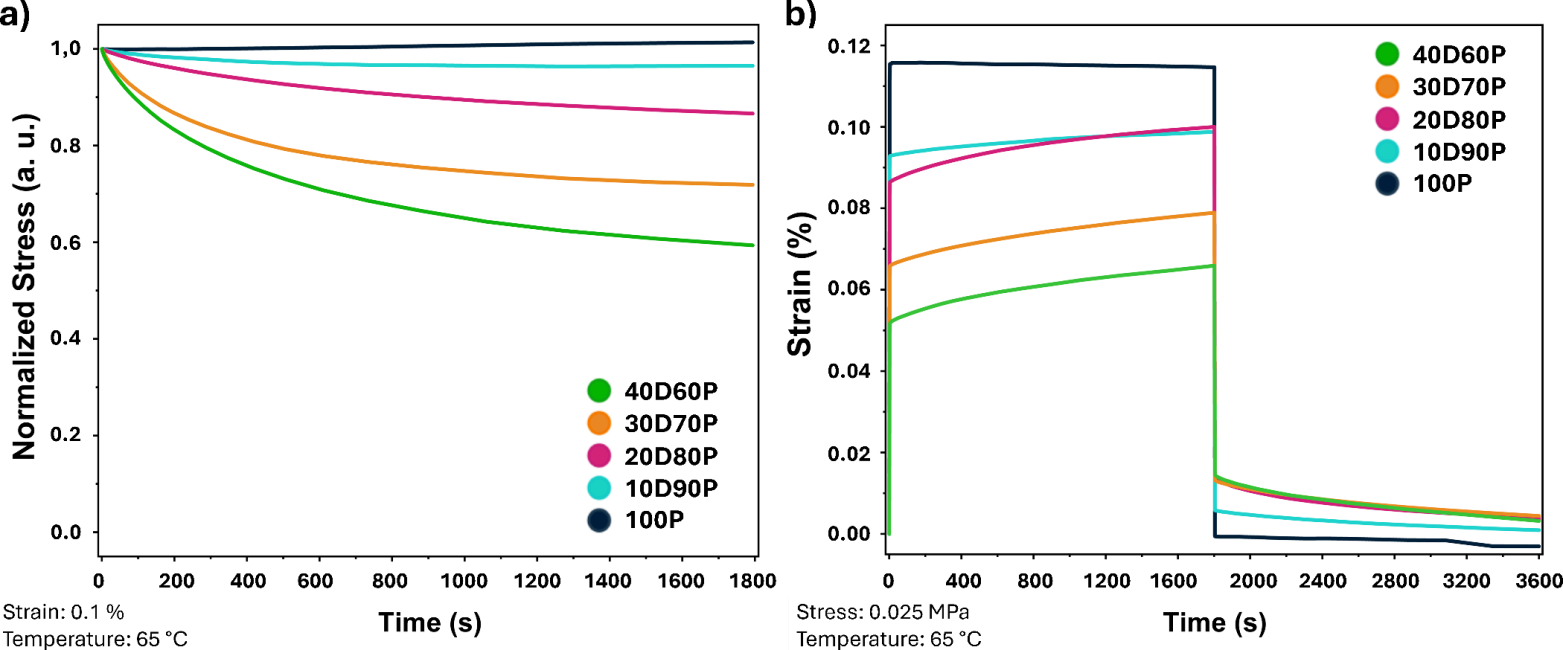
(a) Normalized stress relaxation and (b) creep recovery studies conducted on photocured materials.

Stress relaxation studies were performed on the photocured materials at 65 °C (Figure 4a), a temperature at which all materials exhibit a rubbery plateau, as confirmed by previous DMA characterizations. The results indicate that the ability of the materials to relax stress increases with the molar fraction of dynamic **DBEDMA** compared to static **PPGDA**. Specifically, **DBEDMA**-rich materials show both a higher relaxation rate and lower residual stress after 30 min, whereas sample **100P** exhibits no capacity to relieve applied stress. Notably, stress relaxation increases with increasing **DBEDMA** concentration, indicating an increasing ability to rearrange the three-dimensional network due to the dynamicity of the boronate ester bonds, although complete stress relaxation is never achieved, suggesting that static **PPGDA** does not allow complete network rearrangement at this level of incorporation, i.e. at 60 mol % and above.

Resistance of materials to creep was investigated at the same temperature, by applying a constant stress of 0.025 MPa (Figure 4b). The applied stress value was selected based on the results of stress relaxation experiments to ensure that the instantaneous deformation for all materials remained within the range of 0.01–0.1%. Creep-recovery tests indicate that instantaneous elastic deformation increases with the molar ratio of **PPGDA** to **DBEDMA**, which correlates with the *E*′ modulus values obtained through DMA. Furthermore, **DBEDMA**-rich materials exhibit a greater increase in deformation with time under stress, suggesting that boronate ester metathesis facilitates further deformation through network topological reorganization.

A similar behavior was observed during the recovery phase, where the original dimensions are immediately restored in the **100P** formulation, while recovery is delayed in samples containing **DBEDMA**. In the case of the **100P** formulation, the recovery phase is characterized by a complete restoration of the applied deformation, confirming a more elastic behavior given by a defined and static macromolecular structure, while a low residual strain is observed for materials incorporating the **DBEDMA** cross-linker, indicating a viscous flow in the material compatible with the structural adaptation of the boronate functionalities to the applied stress. This indicates that creep in 3D-printed materials is likely to be a minimal concern.

### 3.3 VP 3D Printing

Among the photoresin formulations studied, **40D60P** demonstrated an optimal balance, requiring a moderate amount of synthetic **DBEDMA** while effectively relieving applied stress through topological rearrangement. Due to these advantages, this formulation was selected for further 3D printing tests to produce tensile specimens for comparing the mechanical isotropy of 3D-printed parts made from **40D60P** with those fabricated using the model photoresin **40B60P**.

The model photoresin, **40B60P**, was specifically designed to closely resemble **40D60P**, with the dynamic **DBEDMA** cross-linker replaced by an equivalent molar amount of nondynamic bisphenol A ethoxylate dimethacrylate (**BAETDMA**) (see molecular structures and formulation details in Figure 1a and Table 1). **BAETDMA** was chosen for its structural similarity to **DBEDMA**, as both feature a central rigid aromatic core with two flexible side chains terminated by methacrylate groups. The molar mass of **BAETDMA**, determined by ^1^H NMR analysis (Figure S7, using eqs S3 and S4), is 498 g/mol, which is nearly identical to that calculated for **DBEDMA** (498.19 g/mol). These similarities are expected to result in comparable photocuring kinetics and mechanical properties for both **40D60P** and **40B60P**.

Tensile test specimens were 3D printed from both photoresins in two orientations: at 0° and 90° relative to the printing base (as shown in Figure S13). The printing process involved sequential irradiation of 0.07 mm-thick layers, each exposed for 15 s.

In **VP** 3D printing, it is common practice to retain some unreacted functional groups in freshly printed (commonly referred as “green”) samples, which can then react during postcuring improve layer adhesion and material isotropy.

**Figure 5:**
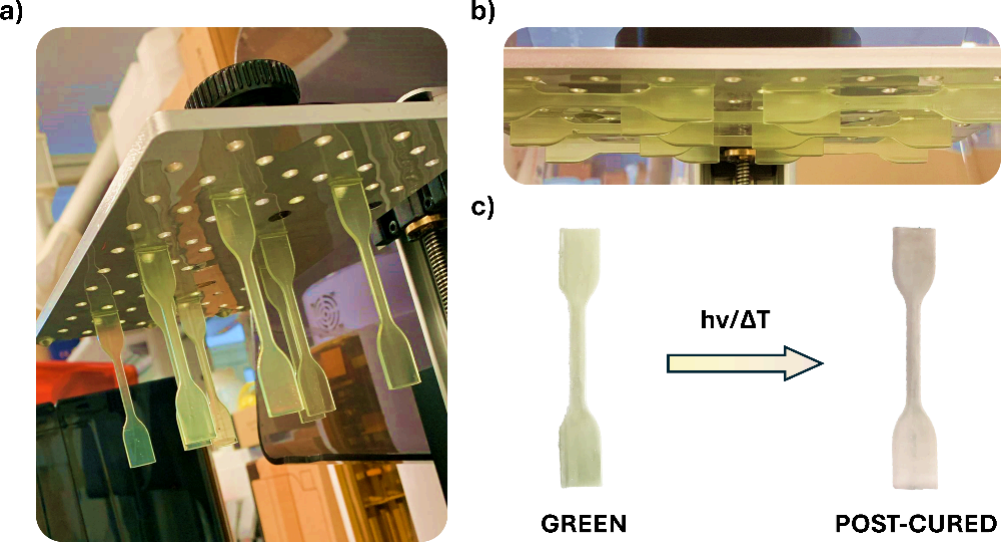
Images of the freshly prepared tensile test specimens printed at (a) 90° and (b) 0° orientations, and (c) color change of green specimens after postcuring.

FTIR analyses (Figures S15 and S16) confirmed that the green tensile specimens (Figures 5a,b) contained a moderate amount of unreacted (meth)acrylates. Postcuring under conditions like those used for photocuring in mold (365 nm monochromatic lamp, 55 mW/cm^2^, 5 min) resulted in a noticeable amount of still unreacted functional groups. However, an alternative postcuring method, using a 405 nm monochromatic lamp for 60 min combined with heating at 80 °C, resulted in a more efficient conversion of the remaining reactive groups, as evidenced by a dramatic change in the color of the samples (Figure 5c). Furthermore, thermal treatment is expected to enhance the rate of boronate ester metathesis, facilitating topological rearrangement. The residual unreacted functional groups were quantified using FTIR spectroscopy on specimens derived from the model photoresin 40B60P, as its spectra enable easier analysis compared to those obtained from 40D60P-derived specimens (refer to FTIR spectra in Figures S14–S16).

### 3.4 Tensile Tests

Since postcuring processes are widely applied to standard 3D-printed materials, tensile tests were conducted exclusively on postcured specimens to evaluate the resulting changes in mechanical anisotropy.

**Figure 6:**
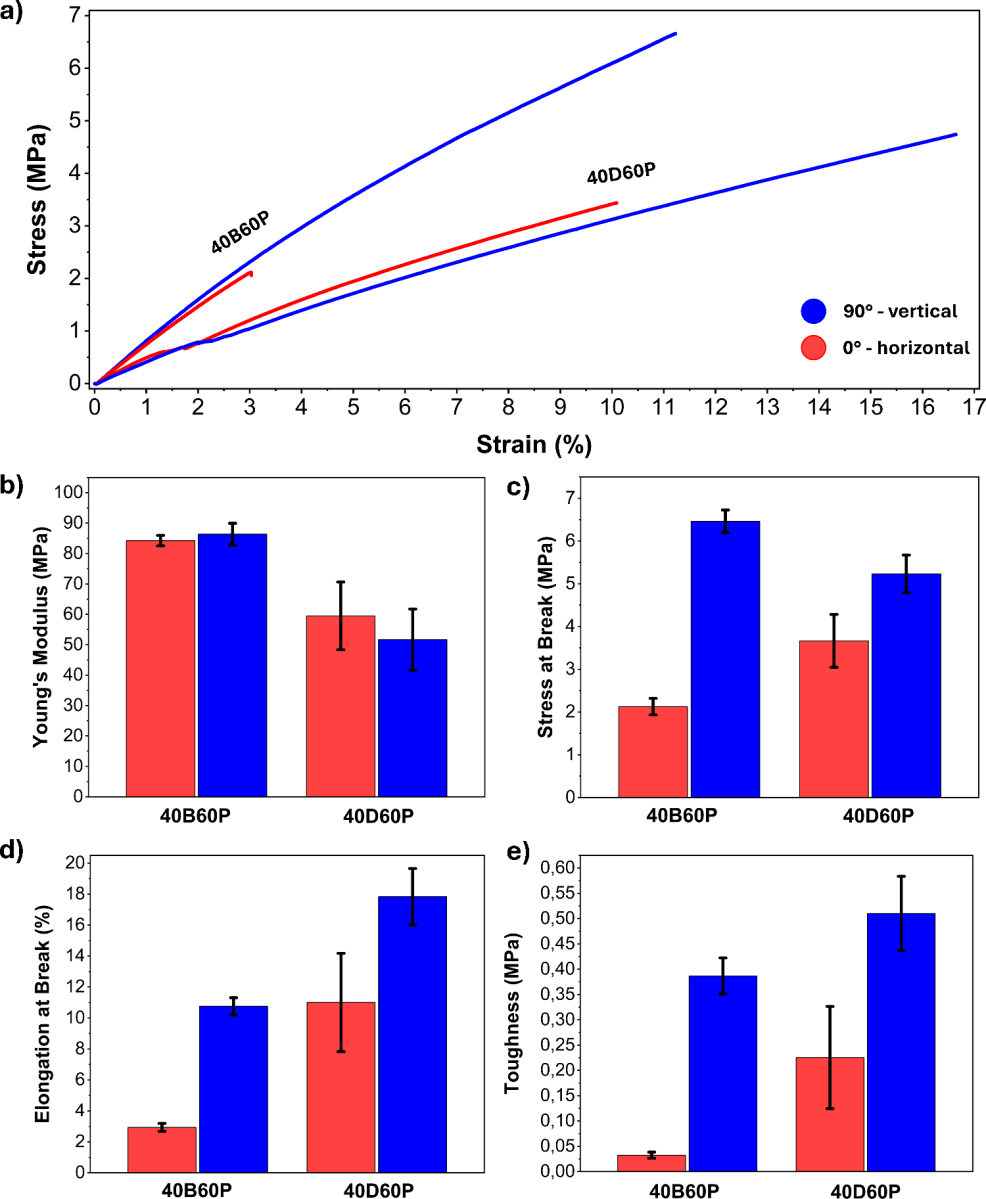
(a) Comparison of representative stress–strain curves for tensile specimens printed with **40B60P** and **40D60P** in 90° (blue) and 0° (red) orientations. Comparison of (b) Young’s modulus, (c) stress at break, (d) elongation at break, (e) and toughness for tensile specimens printed with **40B60P** and **40D60P** in 90° (blue) and 0° (red) orientations.

Notably, samples printed with the model **40B60P** resin exhibited significant differences in tensile tests when comparing samples oriented at 90° to those oriented at 0°. In contrast, samples printed with **40D60P** demonstrated more consistent behavior (Figure 6a).

Additionally, the results indicate that samples prepared from **40D60P** photoresin exhibit lower Young’s modulus but greater toughness than specimens printed with **40B60P** in the same orientation (Figure 6b–e). This difference can be attributed to the presence of **DBEDMA** in **40D60P**, which enables partial topological rearrangement of the polymeric network under applied stress, as confirmed by stress relaxation tests. This rearrangement, which occurs rapidly even at room temperature and with kinetics compatible with tensile test experiments,^36^ is responsible for the increased energy dissipation observed during tensile loading.

**Figure 7:**
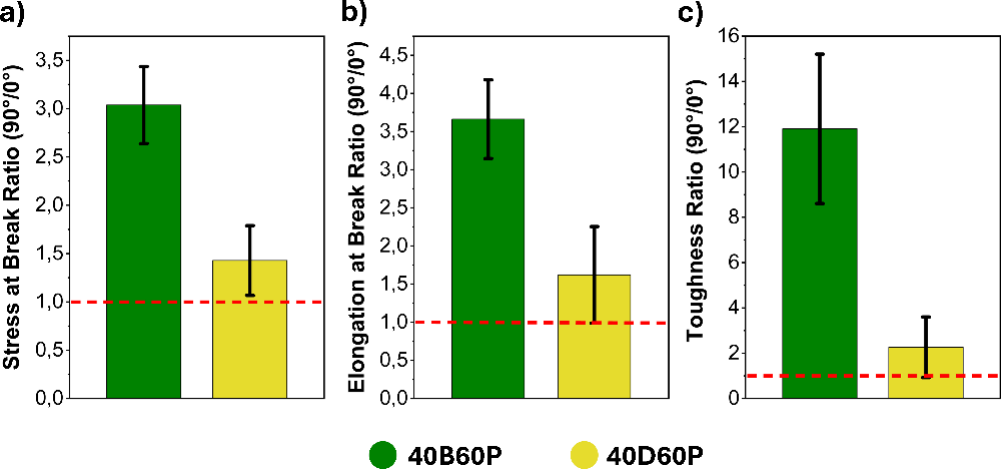
Plotted ratios of (a) stress at break, (b) elongation at break and (c) toughness for samples printed at 90° relative to those printed at 0°.

The parameters exhibiting the greatest variation in materials synthesized with **40B60P** are stress at break, elongation at break, and consequently toughness. Specifically, the 90° oriented samples exhibited stress and elongation at break values approximately 3–4 times greater than those of the 0° samples (Figure 6c,d), while their toughness was nearly 12 times higher (Figure 7). Conversely, no significant difference in Young’s modulus was observed between the two orientations.

This disparity may be attributed to the variation in internal stresses accumulated within the printed specimens, which are greater with wider layer surface areas due to increased shrinkage during photopolymerization. Shrinkage during photopolymerization creates internal stresses that make the material susceptible to warping as it tries to relieve these stresses.^46^ Specimens printed in the 0° orientation have fewer layers, but each with a larger surface area, compared to those printed in the 90° orientation. Considering that the curl deformation is proportional to the length of the raster pattern,^47^ this configuration leads to a greater accumulation of internal stresses, which consequently results in reduced toughness and lower mechanical performance at failure.

Similar behaviors were observed in the specimens printed with the **40D60P** photoresin. However, in this case, the increases in stress at break, elongation at break, and toughness for the 90° samples compared to the 0° samples are significantly less pronounced. Specifically, the stress and elongation at break values for the 90° samples are approximately 1.5 times higher, while toughness is only doubled (Figure 7). As with the previous photoresin, no significant differences in Young’s modulus were found between the two orientations. The lower anisotropy of specimens printed using the **40D60P** formulation, compared to those printed with the **40B60P** formulation, is explained by their ability to relax part of the internal stress accumulated during 3D printing, as well as by the formation of additional covalent bonds between adjacent printed layers. This occurs both through postpolymerization of the material (enabled by sample irradiation during postcuring) and through dynamic bond rearrangement (occurring at room temperature and during heating in the postcuring process).

The specimens printed with the **40D60P** photoresin cannot be classified as fully isotropic, as the measured parameter values for the 90° and 0° samples do not coincide within the margin of error. This limitation is attributed to the substantial degree of static cross-linking, which significantly constrains the topological rearrangement permitted by the dynamic cross-linker. Nevertheless, the enhancements in mechanical anisotropy are remarkable in comparison to a fully static network.

**Figure 8:**
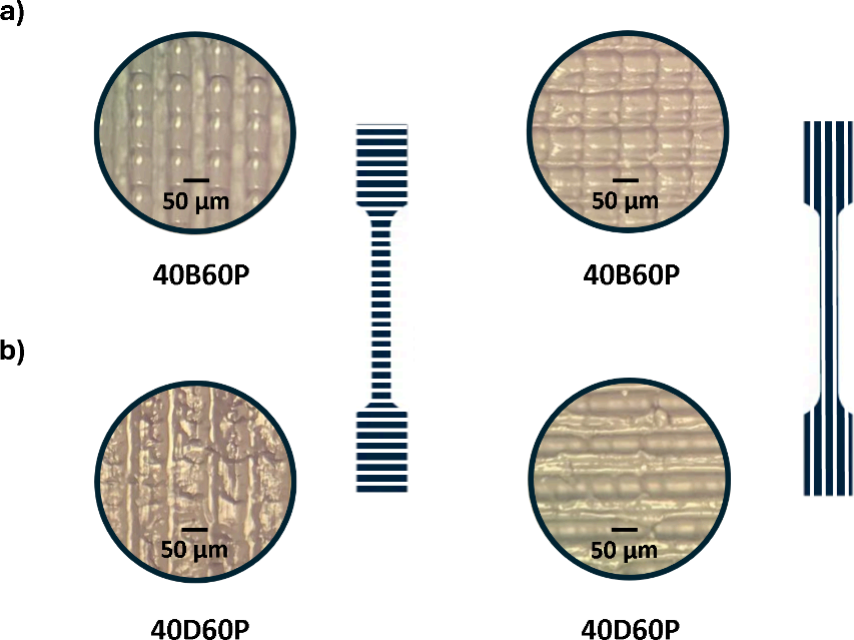
Optical microscopy images of postcured 3D-printed samples using (a) **40B60P** and (b) **40D60P**, shown in both 90° (left) and 0° (right) orientations.

Optical microscopy images of samples 3D-printed with **40B60P** photoresin reveal a characteristic grid pattern often observed in **VP** 3D-printed materials,^48^ with each repetitive unit of the grid displaying a parabolic-shaped motif (see Figure 8a). This pattern forms during the layer-by-layer curing process intrinsic to **VP** 3D printing. Each layer is cured individually and sequentially by the two-dimensional array of square pixels on the LCD screen, with each pixel contributing to a single parabolic-shaped motif.^48^ The images clearly show that the surface pattern is more pronounced in samples obtained from static **40B60P**, whereas samples produced from the partially dynamic **40D60P** photoresin exhibit a smoother, yet still distinct, surface pattern, suggesting partial network rearrangement (see Figure 8b).These observations are consistent with tensile test results. The attenuation of the surface pattern is associated with material homogenization, resulting from partial topological rearrangement and leading to reduced mechanical anisotropy.

## 4 Conclusions

In conclusion, this study demonstrates the successful incorporation of dynamic boronate ester cross-links into photoresins that undergo free radical polymerization of (meth)acrylates, enabling their use in **VP** 3D printing applications. The photoresins exhibited rapid photocuring kinetics, with the curing rate increasing as the molar percentage of acrylates to methacrylates increases. The photopolymerized materials exhibited a remarkable ability to relax some of the applied stress while maintaining resistance to creep, indicating that 3D-printed objects exhibit dimensional stability despite the presence of dynamic boronate ester cross-links in their structure. Additionally, the materials showed a significant reduction in the mechanical anisotropy typically observed in 3D-printed structures, although they cannot yet be classified as fully isotropic. The reduction in mechanical anisotropy can be attributed to the partial topological rearrangement enabled by boronate ester metathesis. This rearrangement is further supported by the partial surface smoothing observed through optical microscopy. These findings highlight the potential of dynamic boronate esters to enhance the performance of 3D-printed materials, paving the way for further advancements in the design and fabrication of high-performance polymer networks.

## Supporting Information Available

Synthesis and characterization of cross-linkers, formulation and photocuring of resins details, photorheology, DSC, FTIR, and TGA analyses; and 3D printing and postcuring of the specimens (PDF)
