# Supplementary material for: Boronate Esters Dynamic Networks for the Reduction of Mechanical Anisotropy in Vat 3D Printed Manufacts
Source: ACS Appl Polym Mater. 2025 Feb 11;7(4):2624–32. doi: 10.1021/acsapm.4c04101 (PMC11877497; doi:10.1021/acsapm.4c04101)
Supplement: Supplementary file 1 — ap4c04101_si_001.pdf [file ap4c04101_si_001.pdf]

Supporting information

Boronate Esters Dynamic Networks for the Reduction of Mechanical Anisotropy in Vat 3D Printed Manufacts

Alex Bonacini[a], Elena Saccani[b], Corrado Sciancalepore[b], Daniel Milanese[b], Gabriele Drago[c], Alessandro Pedrini[a], Roberta Pinalli[a], Renaud Nicolaÿ[d], and Enrico Dalcanale[a]*.*

[a] Department of Chemistry, Life Sciences and Environmental Sustainability, University of Parma, Parco Area delle Scienze 17/A, 43124, Parma, Italy.
[b] Department of Systems and Industrial Technologies Engineering, University of Parma, Parco Area delle Scienze 181/A, Parma 43124, Italy.
[c] ELANTAS Europe S.r.l., Via San Martino 6, Alessandria, 15028, Italy.
[d] Chimie Moléculaire, Macromoléculaire, Matériaux, ESPCI Paris, Université PSL, CNRS, 75005 Paris, France.

Corresponding Authors

Renaud Nicolaÿ – Email: renaud.nicolay@espci.psl.eu;

Enrico Dalcanale – Email: enrico.dalcanale@unirp.it;

Table of Contents:

1 Diboronate ester dimethacrylate (DBEDMA) synthesis ***Pag. S3 – S4***

2 Diboronate ester dimethacrylate (DBEDMA) NMR characterization ***Pag. S5 – S6***

3 Poly(propylene glycol) diacrylate (PPGDA) NMR characterization and average molar mass calculation ***Pag. S8***

4 Bisphenol A polyethylene glycol diacrylate (BAETDMA) NMR characterization and average molar mass calculation ***Pag. S9***

5 Formulation and photocuring of the resins ***Pag. S10***

6 Photo rheology experiments ***Pag. S11***

7 Thermogravimetric analysis (TGA) ***Pag. S12***

8 Dynamic mechanical analysis (DMA) ***Pag. S13***

9 Differential scanning calorimetry (DSC) ***Pag. S14***

10 VP 3D printing and post-curing of the specimens ***Pag. S15***

11 Fourier transform infrared spectroscopy measurements ***Pag. S16 – S18***

12 Tensile ***Pag. S19***

1 Diboronate ester dimethacrylate (DBEDMA) synthesis

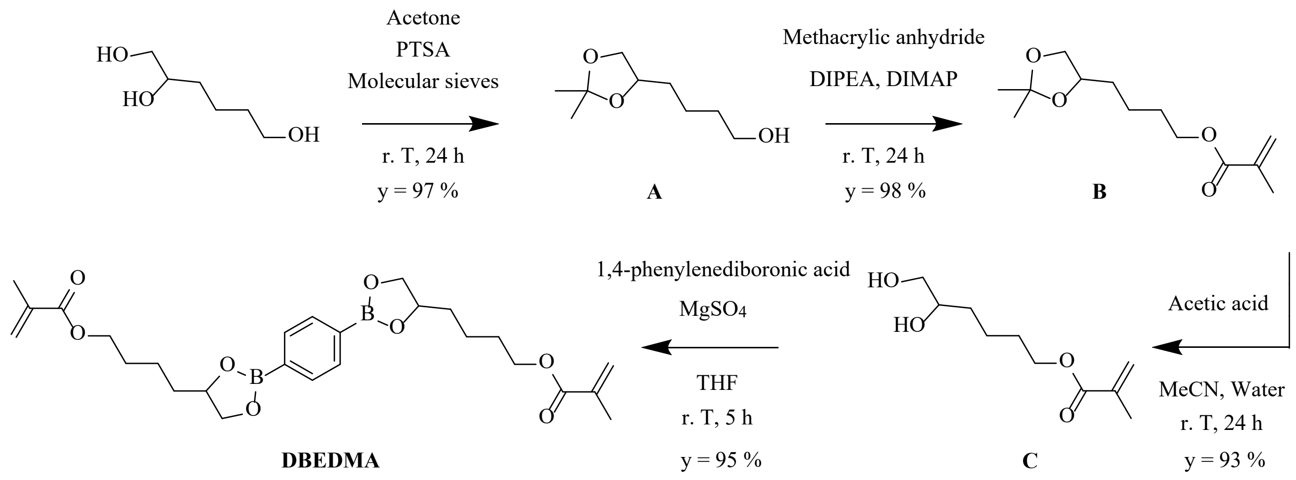

Scheme S1. Synthetic pathway for **DBEDMA**.

All chemical shifts (δ) are reported in parts per million (ppm), referenced either to the residual proton resonances from incomplete deuteration of the NMR solvents or to the solvent's ^{13}C resonance. The abbreviations: s, d, t and m indicated the spectrum peaks referred to: singlet, doublet, triplet and multiplet, respectively. The coupling constant (J) are expressed in Hz.

Compound A

p-Toluenesulfonic acid (5.10 g, 29.7 mmol) and 4 Å molecular sieves (5.60 g) were added to a solution of 1,2,6-hexanetriol (40.2 g, 300 mmol) in acetone (560 mL). The reaction mixture was stirred at room temperature for 24 h. After this time, $NaHCO_3$ (4.28 g, 50.9 mmol) was added, and the mixture was stirred for an additional 10 min. The molecular sieves were removed by filtration and the resulting solution was then concentrated under reduced pressure. An excess of water was added, followed by multiple extractions with DCM. The combined organic layers were collected and concentrated under reduced pressure, yielding compound **A** as a pale-yellow oil (50.9 g, 292 mmol, 97 % yield).

^{1}**H NMR** ($CDCl_3$, 400 MHz): δ (ppm) = 4.06 (m, 1H, -C**H**-), 4.02 (m, 1H, - $C(CH_3)_2OC\mathbf{H_2}'CH$ -), 3.63 (t, J = 6.5 Hz, 2H, $HOC\mathbf{H_2}CH_2$ -), 3.49 (m, 1H, -$C(CH_3)_2COC\mathbf{H_2}''CH$ -), 1.56 (bm, 6H, $HOCH_2C\mathbf{H_2}C\mathbf{H_2}C\mathbf{H_2}CH$ -), 1.39 (s, 3H, $C(C\mathbf{H_3}')_2O$ -), 1.33 (s, 3H, $C(C\mathbf{H_3}'')_2O$ -).

Compound B

N,N-Diisopropylethylamine (79.6 mL, 457 mmol) and 4-dimethylaminopyridine (507 mg, 4.15 mmol) were added to intermediate **A** (72.4 g, 415 mmol) under stirring. Methacrylic anhydride (74.2 mL, 498 mmol) was then slowly added dropwise over the course of 1 h. The resulting mixture was stirred at room temperature for 24 h. After this period, MeOH (30 mL, 741 mmol) was added, and the mixture was stirred for 1 h. Hexane was then introduced, and the solution was extracted multiple times with water, followed by a 1 M HCl aqueous solution. The organic layer was collected and concentrated under reduced pressure, yielding compound **B** as a yellow oil (98.7 g, 407 mmol, 98 % yield).

^{1}**H NMR** ($CDCl_3$, 400 MHz): δ (ppm) = 6.03 (s, 1H, $C\mathbf{H_2}'=C(CH_3)$ -), 5.48 (s, 1H, $C\mathbf{H_2}''=C(CH_3)$ -), 4.08 (m, 1H, - $C(CH_3)_2OC\mathbf{H_2}'CH$ -), 4.08 (t, J = 6.54 Hz, 2H, - $OC\mathbf{H_2}CH_2$ -), 4.02 (m, 1H, - C**H** -), 3.97 (m, 1H, - $C(CH_3)_2OC\mathbf{H_2}'CH$ -), 3.44 (m, 1H, - $C(CH_3)_2OC\mathbf{H_2}''CH$ -), 1.87 (s, 3H, $CH_2=C(C\mathbf{H_3})$ -), 1.53 (bm, 6H, - $OCH_2C\mathbf{H_2}C\mathbf{H_2}C\mathbf{H_2}CH$ -), 1.39 (s, 3H, $C(C\mathbf{H_3}')_2O$ -), 1.34 (s, 3H, $C(C\mathbf{H_3}'')_2O$ -).

Compound C

Acetic acid (54.2 mL, 947 mmol) was added to a solution of intermediate **B** (49.9 g, 206 mmol) dissolved in a mixture of water (250 mL) and MeCN (250 mL). The resulting solution was stirred at room temperature for 24 h. After this period, a large amount of EtOAc was added, and the mixture was extracted multiple times with a 1 M NaOH aqueous solution, followed by water. The organic layer was then collected and concentrated under reduced pressure, yielding compound **C** as a yellow oil (38.7 g, 191 mmol, 93 % yield).

^{1}H NMR ($CDCl_3$, 400 MHz): δ (ppm) = 6.08 (s, 1H, C**H_2'**=C(CH_3) -), 5.54 (s, 1H, C**H_2''**=C(CH_3) -), 4.14 (t, J = 6.51 Hz, 2H, - OC**H_2**CH_2 -), 3.71 (m, 1H, - C**H** -), 3.64 (m, 1H, - C(CH_3)$_2$OC**H_2'**CH -), 3.44 (m, 1H, - C(CH_3)$_2$OC**H_2''**CH -), 1.92 (s, 3H, CH_2=C(C**H_3**) -), 1.55 (bm, 6H, - OCH_2C**H_2**C**H_2**C**H_2**CH -).

DBEDMA

1,4-Phenylenediboronic acid (13.3 g, 80.0 mmol) and water (0.54 mL) were added to a solution of compound **C** (34.0 g, 168 mmol) in THF (173 mL). The resulting solution was stirred at room temperature for 10 min. After this time, anhydrous $MgSO_4$ (57.8 g, 480 mmol) was added, and the mixture was stirred for an additional 5 h at room temperature. The reaction mixture was then filtered, and the filtrate was concentrated under reduced pressure, yielding **DBEDMA** as an orange oil (37.9 g, 76.1 mmol, 95 % yield).

^{1}H NMR ($CDCl_3$, 400 MHz): δ (ppm) = 7.81 (s, 4H, Ar**H**), 6.10 (m, 2H, C**H_2'**=C(CH_3) -), 5.55 (m, 2H, C**H_2''**=C(CH_3) -), 4.59 (m, 2H, - C**H** -), 4.44 (m, 2H, - C(CH_3)$_2$OC**H_2'**CH -), 4.18 (t, J = 6.49 Hz, 2H, - OC**H_2**CH_2 -), 3.95 (m, 2H, - C(CH_3)$_2$OC**H_2''**CH -), 1.94 (s, 6H, CH_2=C(C**H_3**) -), 1.65 (bm, 12H, - OCH_2C**H_2**C**H_2**C**H_2**CH -).

2 Diboronate ester dimethacrylate (DBEDMA) NMR characterization

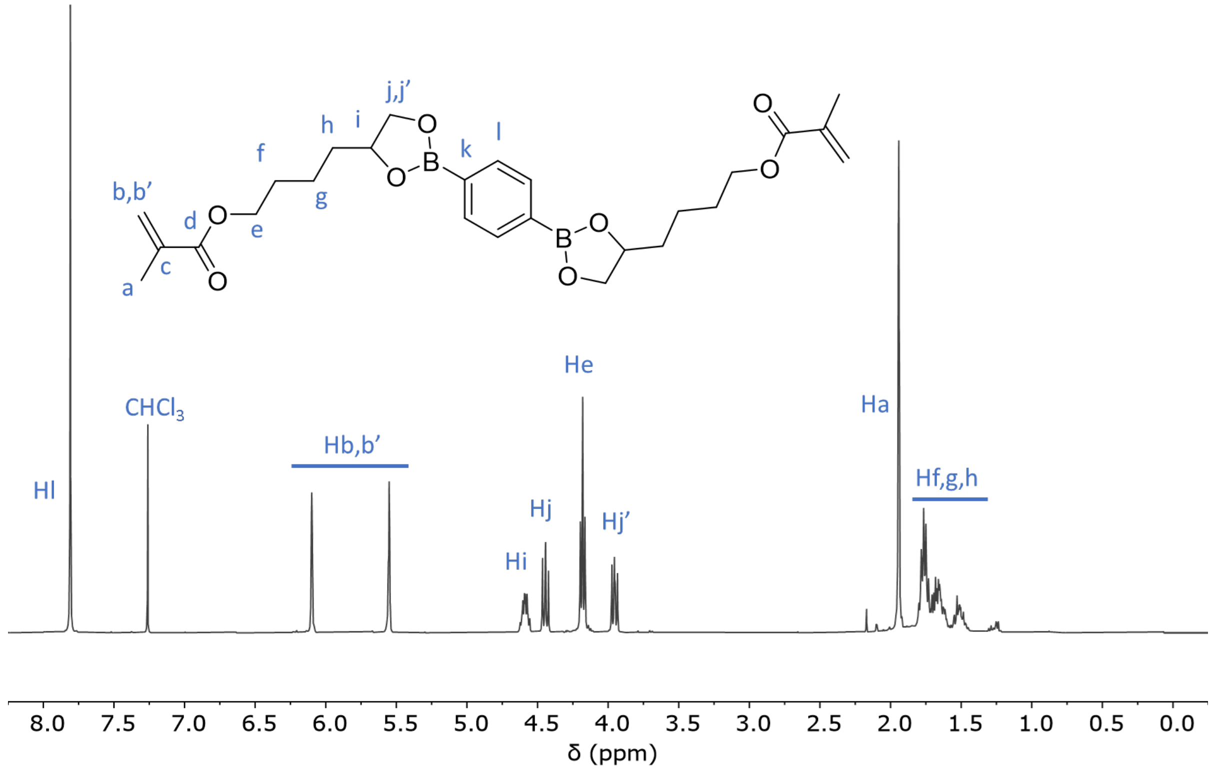

Figure S1. ^{1}H NMR of **DBEDMA**, in $CDCl_3$, 400 MHz, 25 °C.

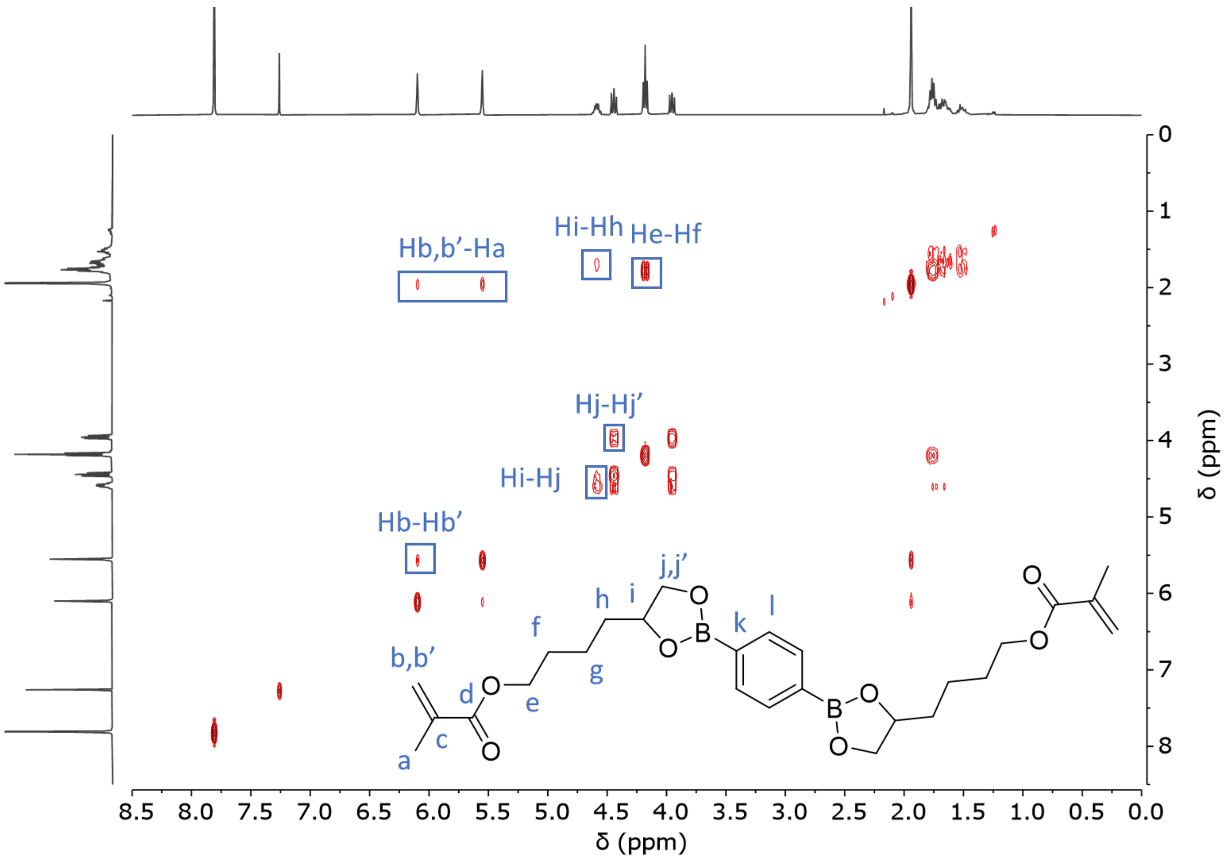

Figure S2. COSY NMR of **DBEDMA**, in $CDCl_3$, 400 MHz, 25 °C.

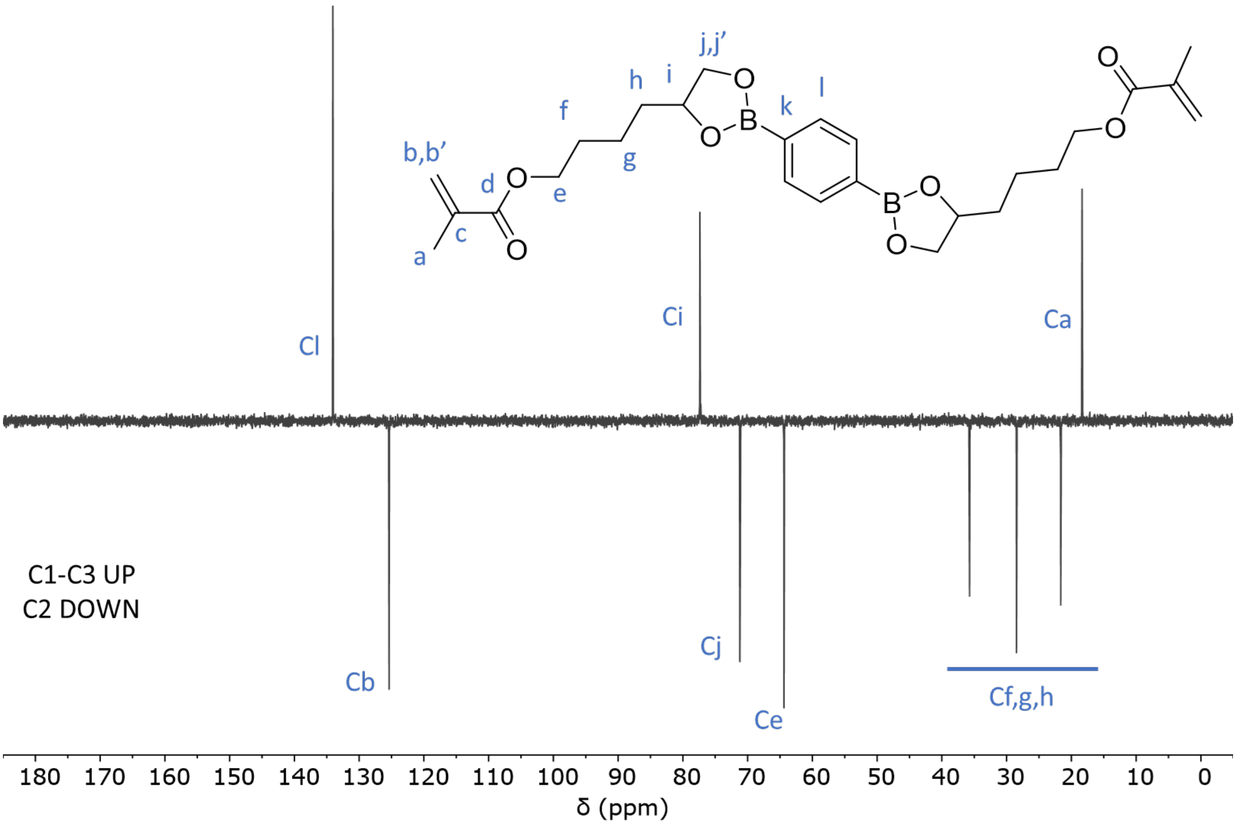

Figure S3. ^{13}C DEPT135 NMR of **DBEDMA** (CH_3 and CH up; CH_2 and C down), in $CDCl_3$, 101 MHz, 25 °C.

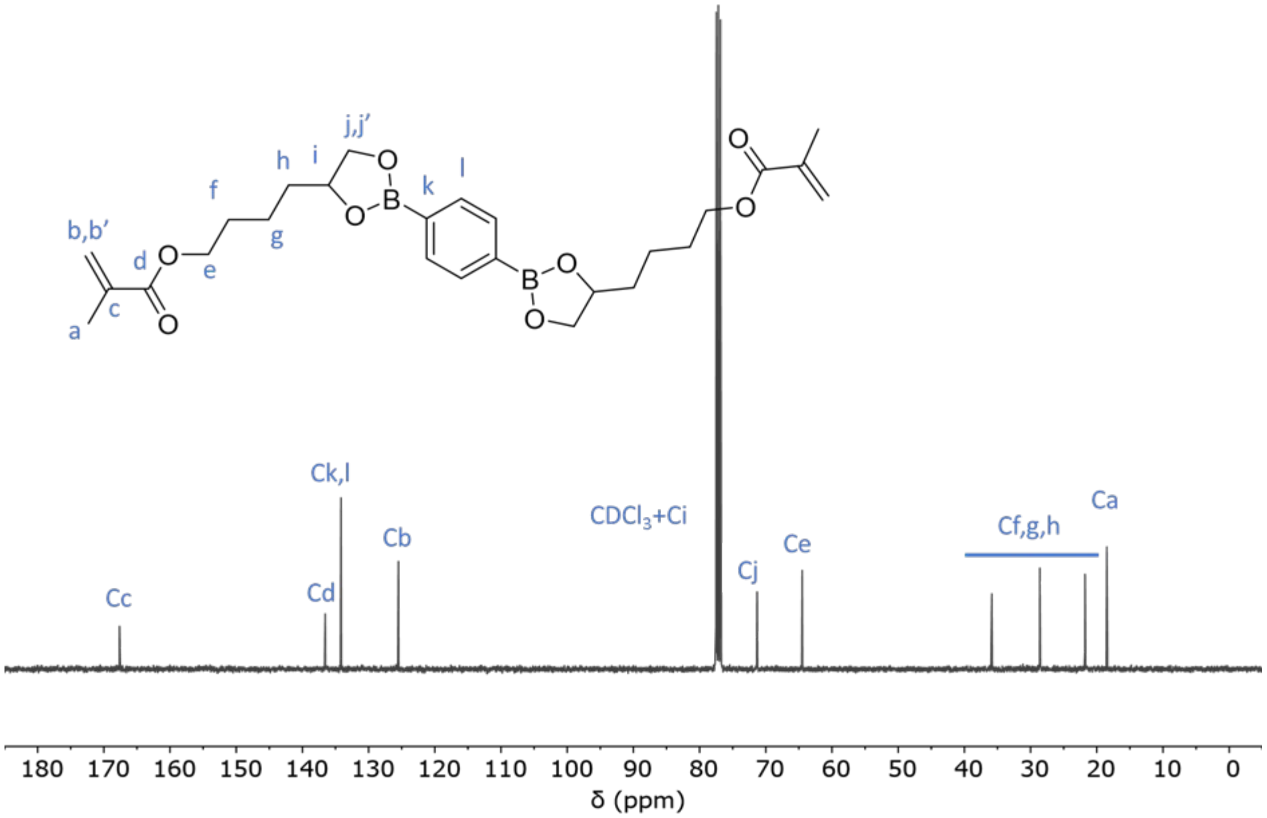

Figure S4. ^{13}C NMR of **DBEDMA**, in $CDCl_3$, 101 MHz, 25 °C.

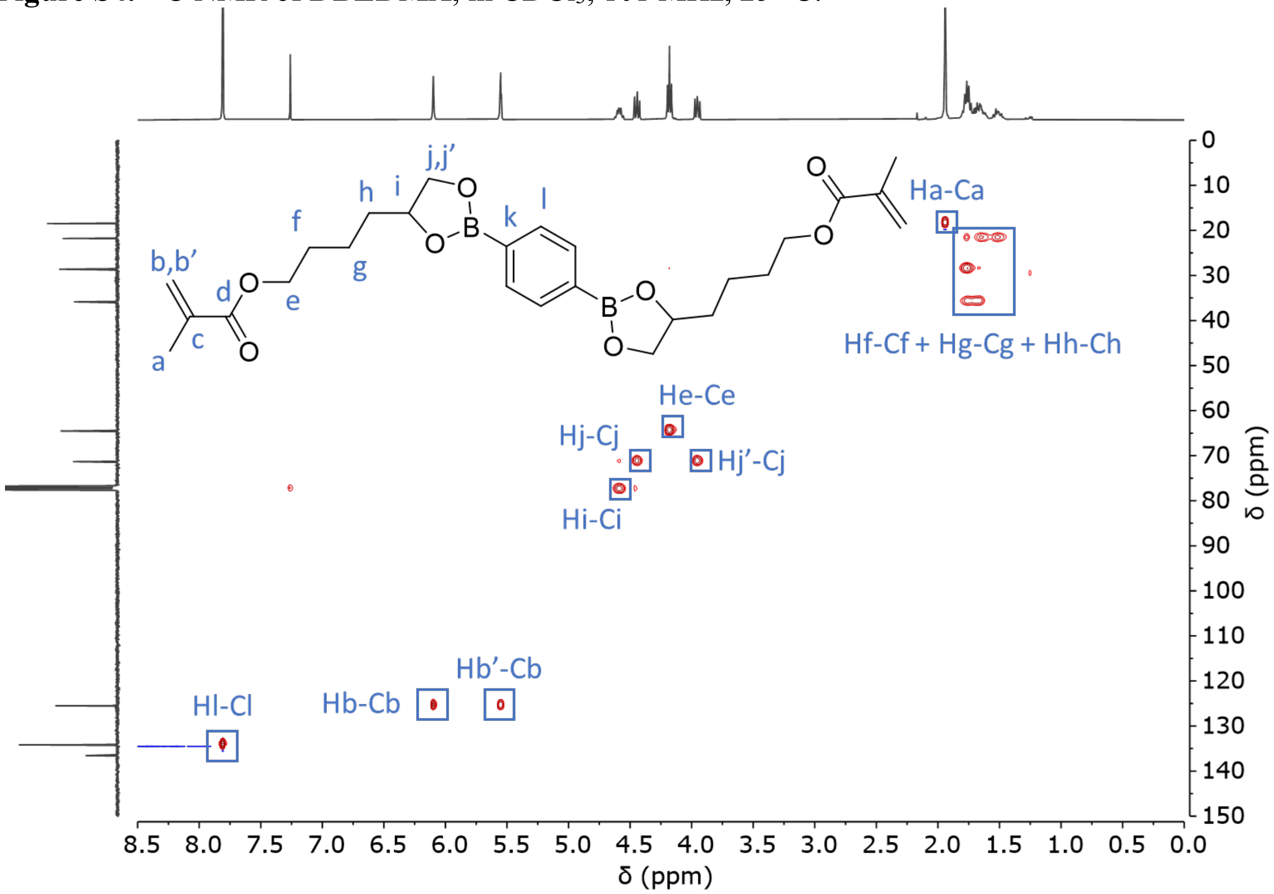

Figure S5. HSQC NMR of **DBEDMA**, in $CDCl_3$, 400 MHz, 25 °C.

3 Poly(propylene glycol) diacrylate (PPGDA) NMR characterization and average molar mass calculation

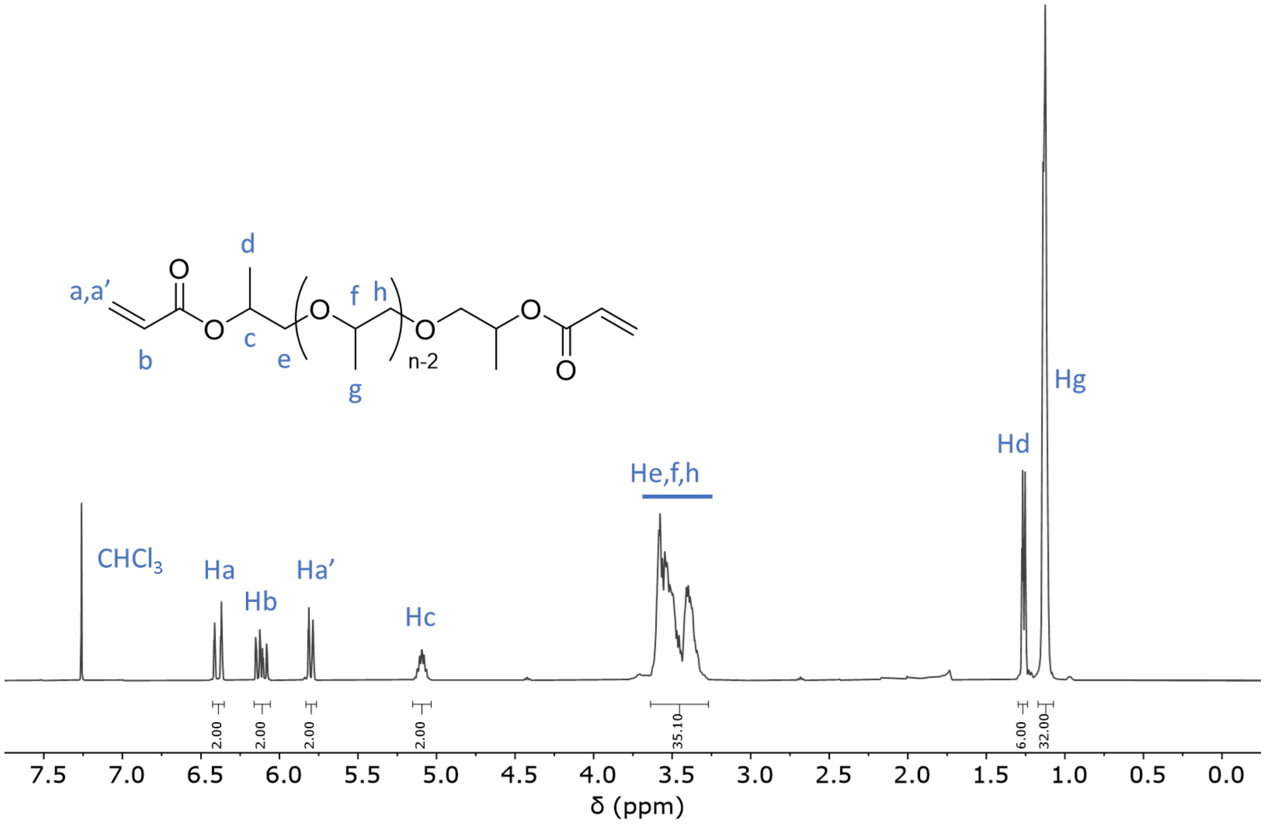

Figure S6. ^{1}H NMR of **PPGDA**, in $CDCl_3$, 400 MHz, 25 °C.

$$\mathbf{n} = \frac{\int \mathbf{Hc} + \int \mathbf{He,f,h} + \int \mathbf{Hd} + \int \mathbf{Hg}}{6} = \mathbf{12.52}$$

Equation S1. Formula for calculating the average **n** value of **PPGDA**.

$$\mathbf{M_w} = [124.14 + \mathbf{n}\ (58.08)]\ \text{g/mol} = \mathbf{851\ g/mol}$$

Equation S2. Formula for calculating the average molecular weight of **PPGDA**.

4 Bisphenol A polyethylene glycol diacrylate (BAETDMA) NMR characterization and average molar mass calculation

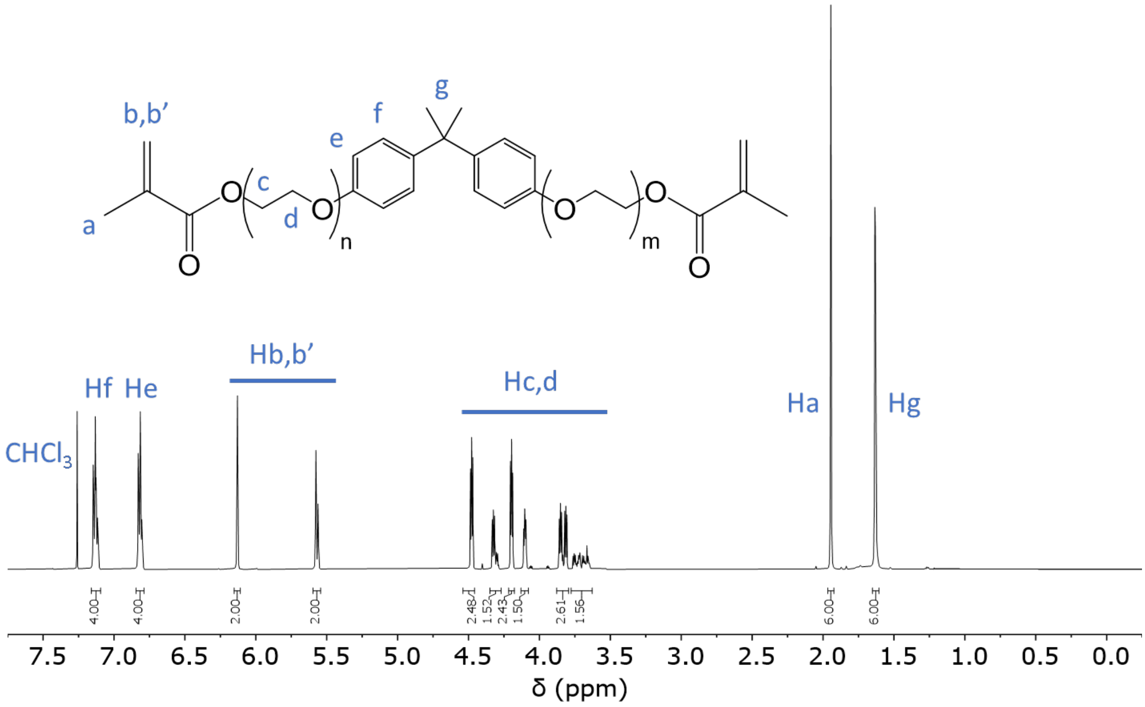

Figure S7. ^{1}H NMR of **BAETDMA**, in $CDCl_3$, 600 MHz, 25 °C.

$$(\mathbf{n}+\mathbf{m}) = \frac{\int \mathbf{Hc,d}}{4} = \mathbf{3.03}$$

Equation S3. Formula for calculating the average **n + m** value of **BAETDMA**.

$$\mathbf{M_w} = [364.44 + (\mathbf{n}+\mathbf{m})\ (44.05)]\ \mathrm{g/mol} = \mathbf{498\ g/mol}$$

Equation S4. Formula for calculating the average molecular weight of **BAETDMA**.

5 Formulation and photocuring of the resins

	DBEDMA	PPGDA	BAETDMA	BAPO
**100P**	/	11.9 g (14.0 mmol) 100 mol%	/	11.8 mg (0.28 mmol) 2mol%
**10D90P**	0.70 g (2.40 mmol) 10 mol%	10.7 g (12.6 mmol) 90 mol%	/	11.8 mg (0.28 mmol) 2mol%
**20D80P**	1.40 g (2.80 mmol) 20 mol%	9.52 g (11.2 mmol) 80 mol%	/	11.8 mg (0.28 mmol) 2mol%
**30D70P**	2.10 g (4.20 mmol) 30 mol%	8.33 g (9,80 mmol) 70 mol%	/	11.8 mg (0.28 mmol) 2 mol%
**40D60P**	2.79 g (5.60 mmol) 40 mol%	7.14 g (8.40 mmol) 60 mol%	/	11.8 mg (0.28 mmol) 2mol%
**60D40P**	4.19 g (8.40 mmol) 60 mol%	4.76 g (5.60 mmol) 40 mol%	/	11.8 mg (0.28 mmol) 2 mol%
**80D20P**	5.58 g (11.2 mmol) 80 mol%	2.38 g (2.80 mmol) 20 mol%	/	11.8 mg (0.28 mmol) 2 mol%
**100D**	6.98 g (14.0 mmol) 100 mol%	/	/	11.8 mg (0.28 mmol) 2 mol%
**40B60P**	7.14 g (8.40 mmol) 60 mol%	/	2.79 g (5.60 mmol) 40 mol%	11.8 mg (0.28 mmol) 2 mol%

Table S1. Measured masses and moles of each component used to prepare approximately 10 g of each formulation. **DBEDMA** is indicated as **D**, **PPGDA** as **P**, and **BAETDMA** as **B**.

6 Photo rheology experiments

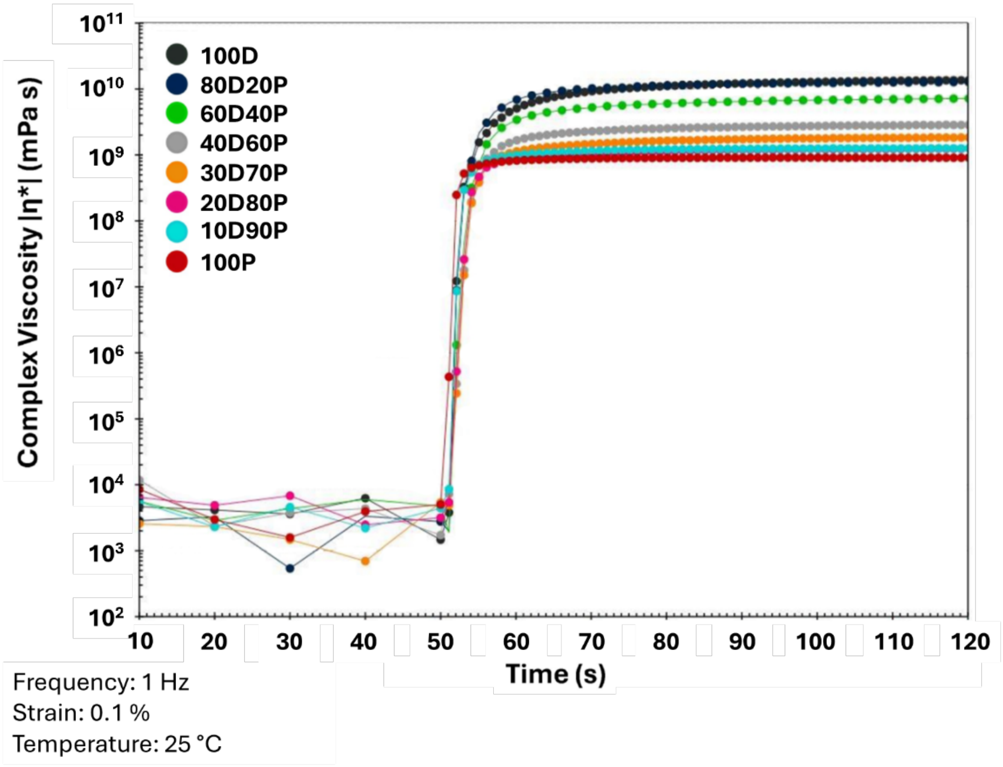

Figure S8. Complex viscosity measured before (from 10 s to 50 s) and during (from 50 s to 120 s) UV light exposure of the photocurable formulations.

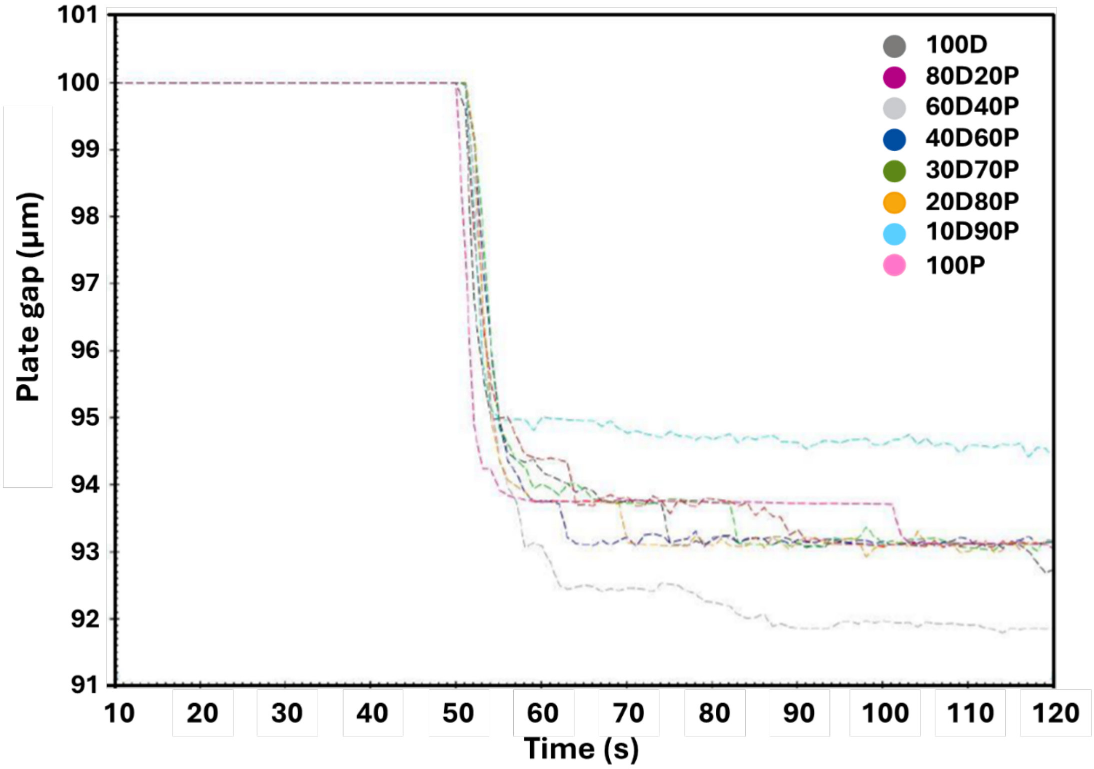

Figure S9. Resin shrinkage measured as a reduction in plate gap as result of UV light exposure (from 50 s to 120 s) of the photocurable formulations, while a constant normal force is applied.

7 Thermogravimetric analysis (TGA)

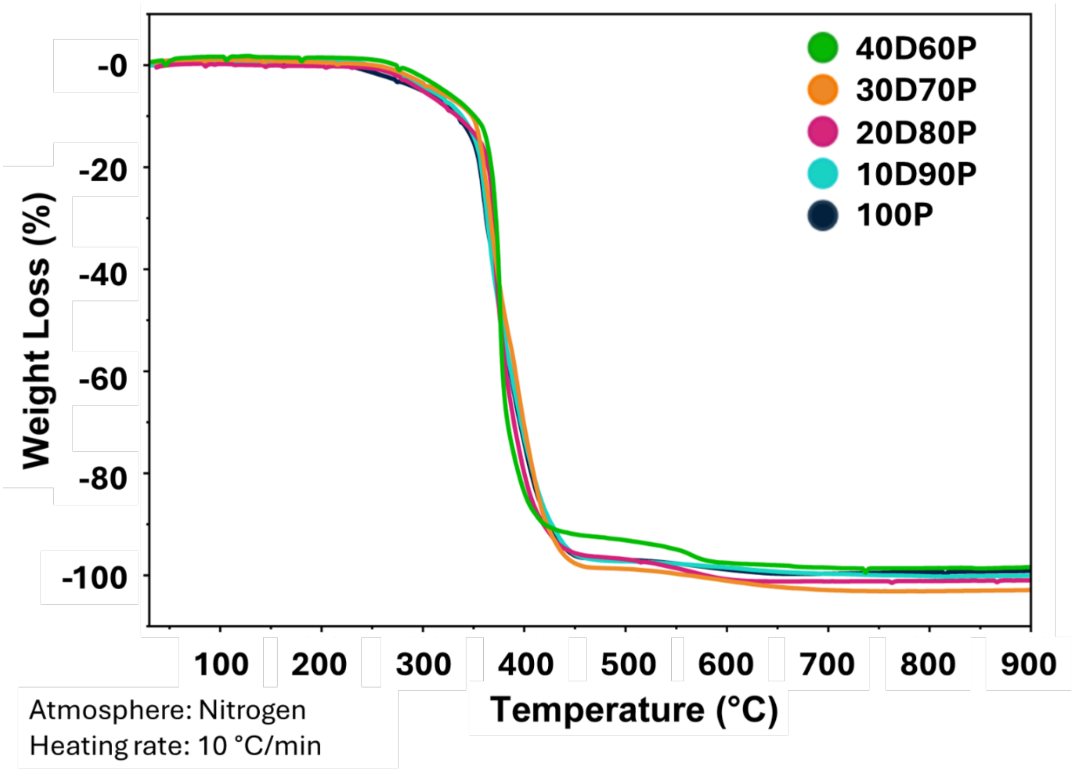

Figure S10. Thermogravimetric analysis of the photocured materials.

8 Dynamic mechanical analysis (DMA)

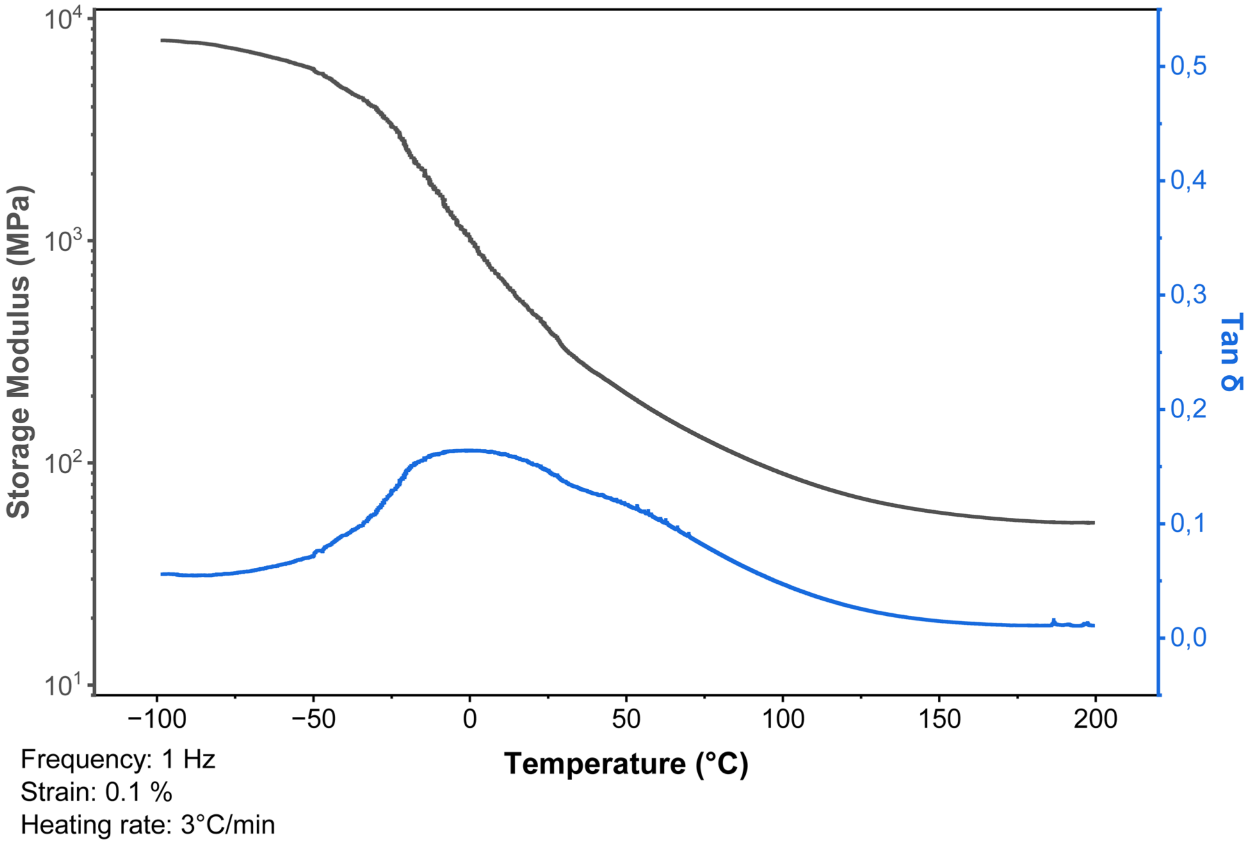

Figure S11. Storage modulus and tan δ of photocured **60D40P** as function of temperature.

9 Differential scanning calorimetry (DSC)

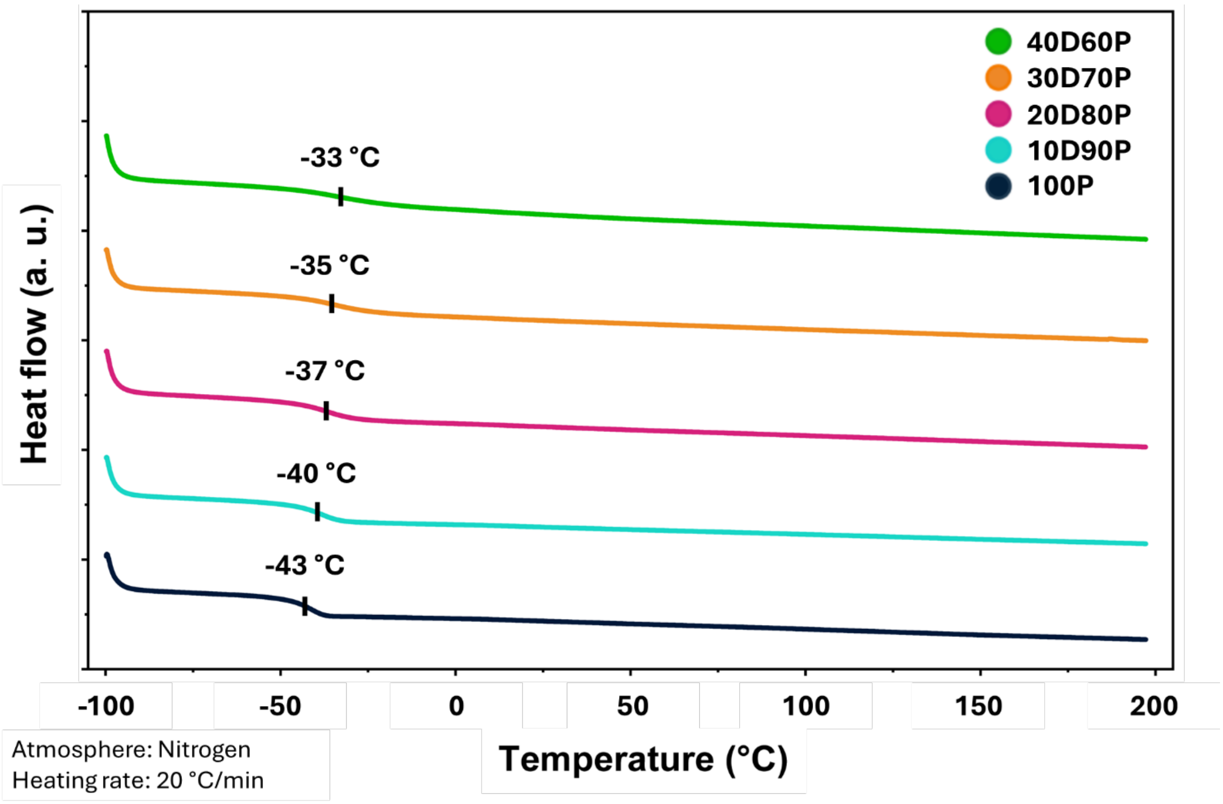

Figure S12. Differential scanning calorimetry analysis of the photocured materials.

10 VP 3D printing and post-curing of the specimens

Both resins, **40D60P** and **40B60P**, were used to 3D print tensile test specimens in two different orientations:

- In the first orientation, the main extension axis is perpendicular to the printing base and parallel to the normal of the sequentially printed layers (90° orientation, **Figure S13a**).
- In the second orientation, the main extension axis is parallel to the printing base and perpendicular to the normal of the material layers (0° orientation, **Figure S13b**).

Eight identical specimens were printed simultaneously in each run, following the arrangements shown below. All the specimens were printed with a layer thickness of 0.07 mm, and each layer was irradiated for a total of 15 s.

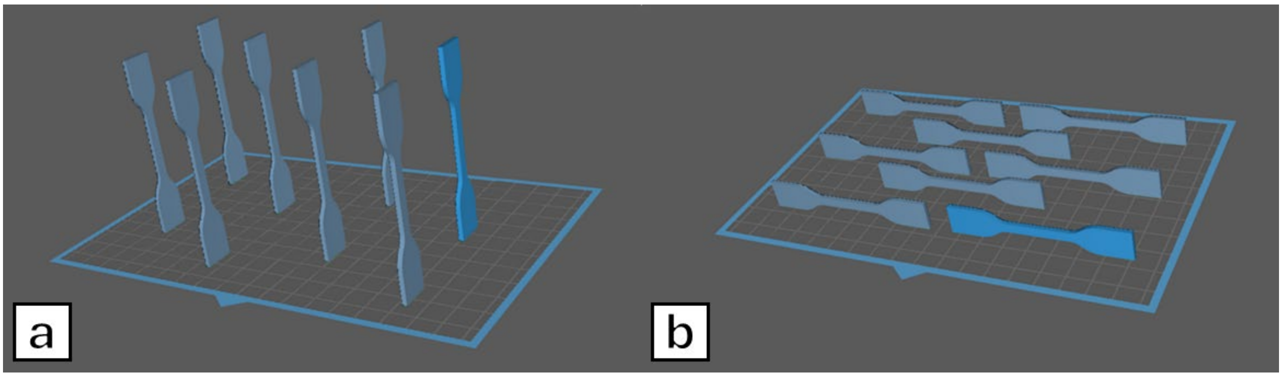

Figure S13. Arrangement of the specimens to be printed at angles of **a)** 90° and **b)** 0°, generated using ChiTuBox software.

For each print, 100 g of photocurable formulation, prepared according to the procedure described above, were used.
At the end of the printing process, the samples are carefully removed from the metal plate and washed with abundant MeCN. The solvent is allowed to evaporate for about an hour while keeping the printed objects shielded from light.

The post-curing of the 3D-printed specimens was performed using two alternative methods:

- In the first method, one of the larger flat surfaces of the sample was irradiated for 5 min using monochromatic light at 365 nm with a power density of 55 mW/cm^2.
- In the second method, the samples were first heated to 80 °C, then one of the larger flat surfaces was irradiated with 405 nm monochromatic light for 1 hour, while maintaining the same temperature.

11 Fourier transform infrared spectroscopy measurements

FTIR spectra in ATR mode were acquired for 3D-printed green specimens fabricated using both the **40D60P** and **40B60P** formulations.

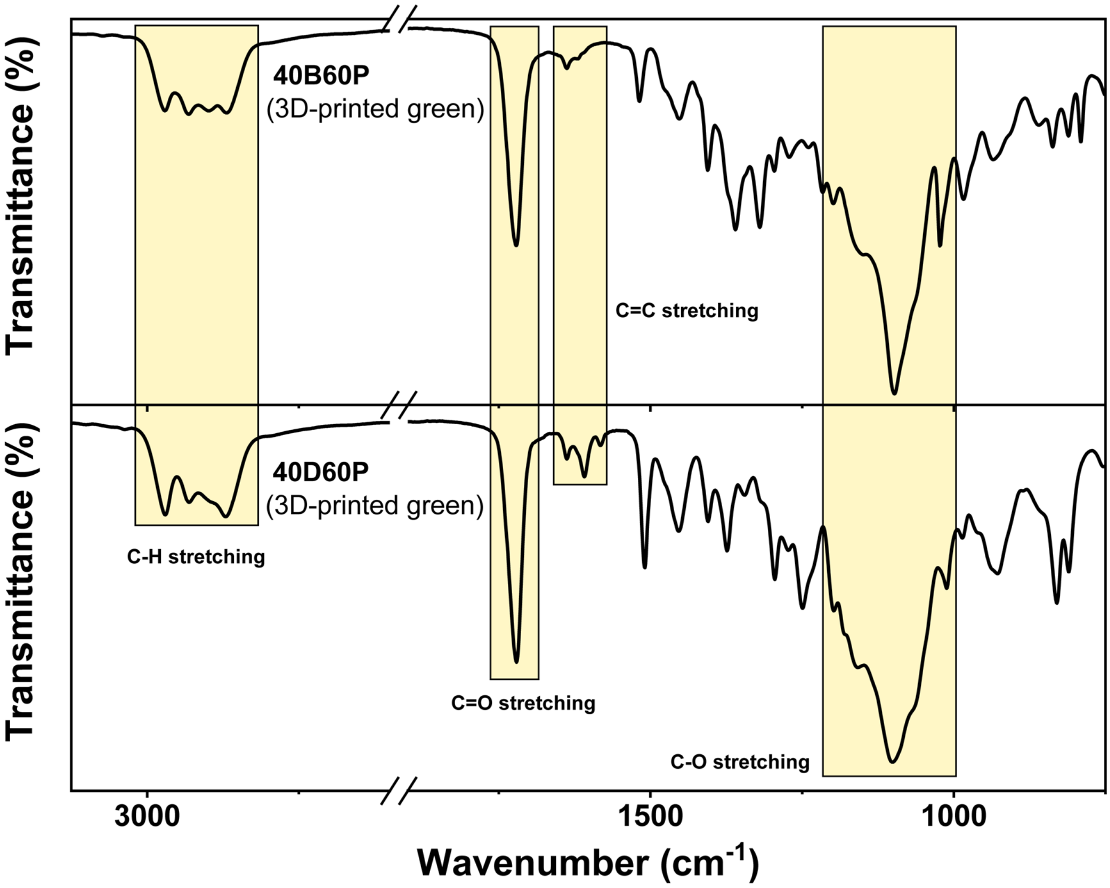

Figure S14. FTIR spectra of 3D printed tensile test specimens in green state obtained from **40D60P** and from **40B60P**.

The FTIR spectra for the **40B60P** formulation and the photocured tensile specimens derived from this formulation, produced either through direct photocuring or via 3D printing followed by post-curing, are shown below.

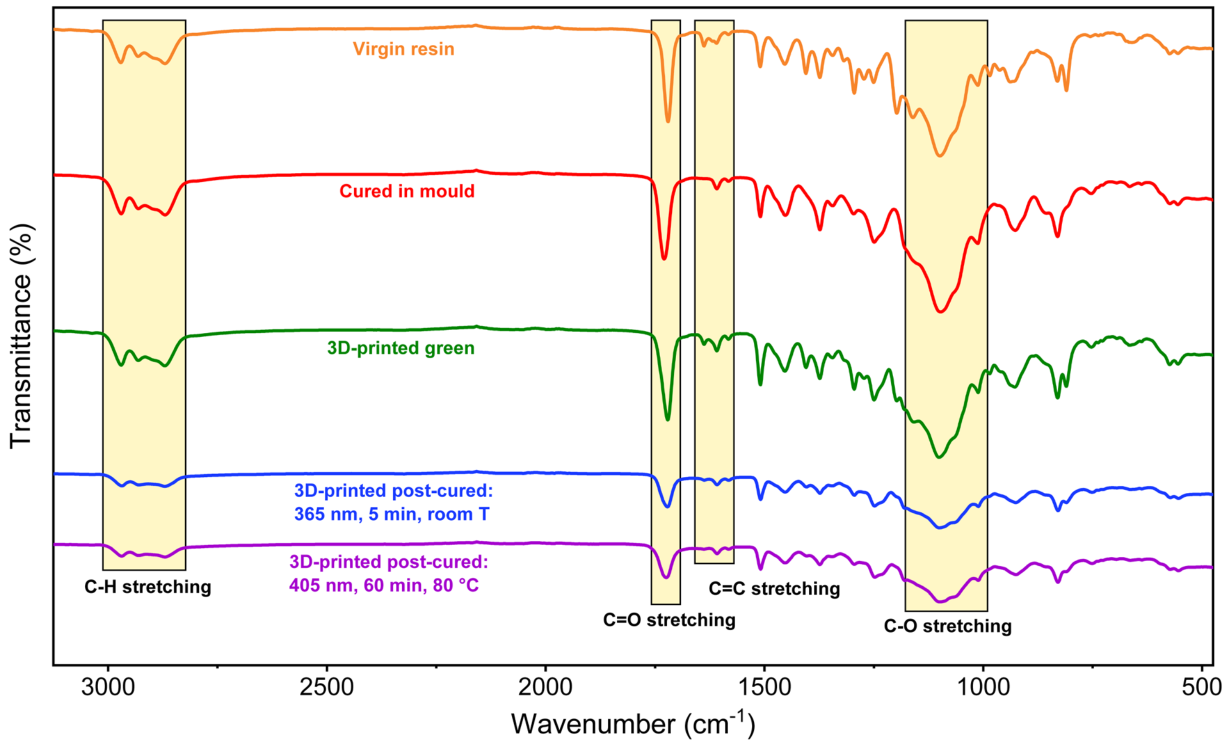

Figure S15. FTIR spectra of tensile test specimens obtained from **40B60P** formulation, displayed in transmittance as a function of wavenumber.

The collected spectra were then converted to absorbance as a function of wavenumber, and a baseline, calculated using a fifth-degree polynomial, was subtracted.

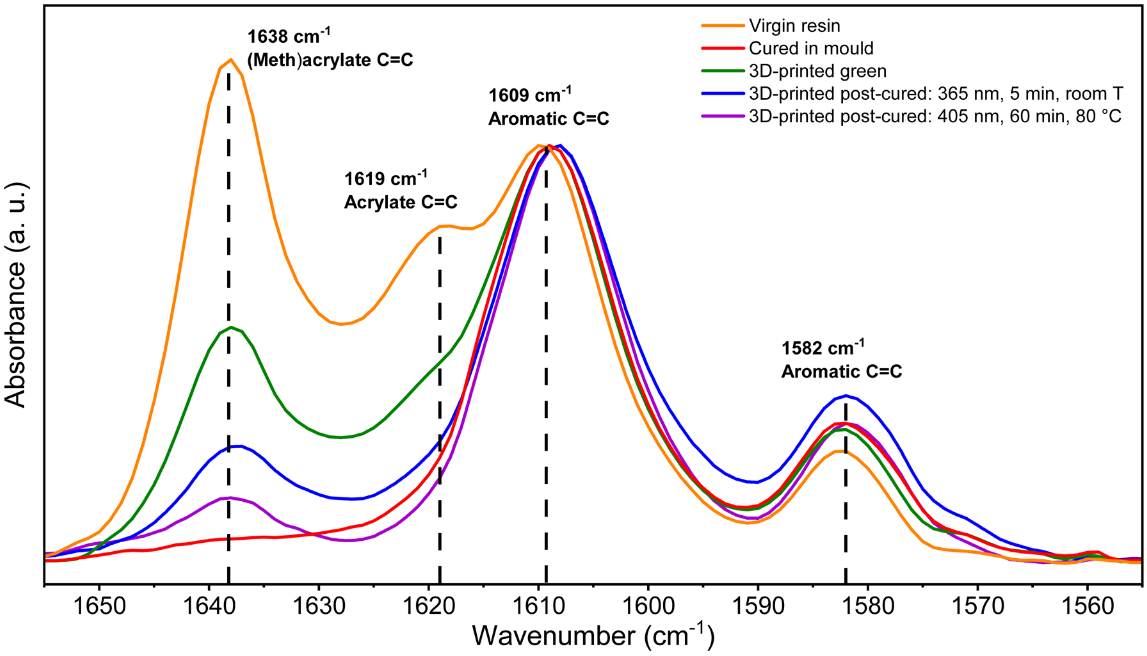

Figure S16. FTIR spectra of tensile test specimens, shown as absorbance versus wavenumber, normalized to the 1609 cm^{-1} peak.

12 Tensile tests

The printed specimens were subjected to mechanical tensile tests using a dynamometer. For each sample, the thickness and width of the central section, as well as the distance between the grips holding the specimen in the dynamometer, were initially measured.
The tensile tests were conducted by applying a pre-tension of up to 2 N at a speed of 5 mm/min, while the actual test was carried out at a tensioning speed of 1 mm/min.
The acquired data were processed into stress-strain curves, from which the toughness, Young's modulus, and the stress and strain at break were calculated for the specimens.
Three replicates of each measurement were taken.
